# Systematic review of the patient burden of generalised myasthenia gravis in Europe, the Middle East, and Africa

**DOI:** 10.1186/s12883-024-03553-y

**Published:** 2024-02-10

**Authors:** J McCallion, A Borsi, W Noel, J Lee, W Karmous, S Sattler, GM Boggia, EJ Hardy, CR Mitchell, SA Mitchell, Nils Erik Gilhus

**Affiliations:** 1 Janssen EMEA, Beerse, Belgium; 2 Mtech Access, Bicester, Oxfordshire UK; 3 Department of Clinical Medicine, University of Bergen, Bergen, Norway; 4 Department of Neurology, Haukeland University Hospital, Bergen, Norway

**Keywords:** Generalised myasthenia gravis, Patient burden, Systematic literature review, Quality of life

## Abstract

**Background:**

Myasthenia gravis (MG) is a rare autoimmune disease characterised by muscle weakness, and progression from ocular (oMG) to generalised (gMG) symptoms results in a substantial negative impact on quality of life (QoL). This systematic review aimed to provide an overview of the patient burden experienced by people living with gMG.

**Methods:**

Electronic database searches (conducted March 2022), supplemented by interrogation of grey literature, were conducted to identify studies reporting patient burden outcomes in patients with gMG in Europe, the Middle East and Africa. Results were synthesised narratively due to the heterogeneity across trials.

**Results:**

In total, 39 patient burden publications (representing 38 unique studies) were identified as relevant for inclusion in the systematic review, consisting of 37 publications reporting formal patient-reported outcome measures (PROMs), and two publications describing alternative qualitative assessments of patient experience. The studies included a variety of measures including generic and disease-specific PROMs, as well as symptom-specific PROMs focusing on key comorbidities including depression, anxiety, fatigue and sleep disturbance. The findings showed some variation across studies and PROMs; however, in general there was evidence for worse QoL in patients with gMG than in healthy controls or in patients with oMG, and a trend for worsening QoL with increasing MG severity.

**Conclusions:**

This review highlights the importance of considering patient QoL when developing and assessing treatment and management plans for patients with gMG. However, the heterogeneity identified across studies illustrates the need for further representative and well-powered studies in large cohorts administering consistent, validated questionnaires.

**Trial registration:**

The protocol for this systematic review was registered in PROSPERO: CRD42022328444.

**Supplementary Information:**

The online version contains supplementary material available at 10.1186/s12883-024-03553-y.

## Background

Myasthenia gravis (MG) is a rare autoimmune neurological disorder, characterised by the presence of pathogenic antibodies that block and damage post-synaptic receptors in the neuromuscular junction, resulting in impairments in neuromuscular transmission and muscle contraction [12, 20, 21, 34]. As a result, patients develop muscle weakness, which can present as a broad range of symptoms including ocular ptosis, diplopia, dysphagia, dysarthria, limb weakness, and respiratory insufficiency [20, 22]. Recent studies in Europe estimate an MG incidence rate of 4–30 cases per million person-years, with prevalence rates ranging between 150–200 cases per million people [20]. MG affects all ages and racial groups, although women are more commonly affected by early-onset MG (< 50 years) than men, and paediatric MG is very rare [12, 20]. Current treatments for MG constitute supportive care, which focuses on improving and managing the symptoms of the disease. Available therapies include acetylcholinesterase inhibitors, immunosuppressive treatments, thymectomy, intravenous immunoglobulins, and plasmapheresis [21, 34, 35]. Monoclonal antibody treatments are increasingly becoming available for MG, including complement (C5) inhibitors (e.g. eculizumab, ravulizumab), neonatal Fc receptor (FcRn) inhibitors (e.g. efgartigimod, nipocalimab, rozanolixizumab), and B cell depleting agents (e.g. rituximab) [2, 34].

When MG patients are diagnosed they most commonly present with ocular symptoms (oMG), with up to 80% of patients going on to develop generalised MG (gMG); typically within two years of disease onset [20]. Patients with gMG experience a wider range of symptoms than patients with oMG and these can be highly unpredictable, potentially manifesting as recurrent exacerbations requiring intervention [20, 22]. In severe cases, patients experience myasthenic crises where mechanical ventilation is required and, in rare cases, may be fatal [12, 21]. The greater symptom burden and risk of exacerbations experienced by people with gMG compared with oMG suggest that this group have a reduced quality of life (QoL).

To our knowledge, there is no published systematic review that focuses specifically on MG patients experiencing generalised symptoms. The objective of this systematic literature review (SLR) was to identify and summarise evidence relating to patient burden in studies of gMG conducted in Europe, the Middle East and Africa.

## Methods

A systematic literature search was performed to identify studies evaluating patient and economic burden in patients with generalised MG in Europe, the Middle East and Africa (EMEA). The study was conducted in accordance with Preferred Reporting Items for Systematic Reviews and Meta-Analyses (PRISMA) guidelines [39]. The protocol for the review was registered in International Prospective Register of Systematic Reviews (PROSPERO) on 3rd May 2022 (CRD42022328444).

Electronic searches of the following databases were conducted on 29th March 2022 via the OVID platform: Embase, Medline®, Medline® Daily, Medline® Epub Ahead of Print (In-Process & Other Non-Indexed Citations), Evidence-Based Medicine Reviews, and EconLit. The full search strategy is provided in the Supplementary information. Additional keyword searches were conducted of relevant congress proceedings from the past three years, rare disease and MG-specific advocacy group websites, the University of Sheffield ScHARRHUD utility database, and Google Scholar. The reference lists of eligible studies were also reviewed to identify any further relevant publications that were not already included.

Records were eligible for inclusion if they reported on real-world evidence conducted in patients with gMG. Studies reporting on a mixed MG population were excluded if results for gMG were not reported separately from oMG and the overall proportion of gMG patients in the population was < 80%. Full eligibility criteria are provided in Table 1. Two independent reviewers screened the title and abstract of citations against the pre-defined inclusion/exclusion criteria. This approach is aligned with published guidance [14, 42]. The full texts of citations included at this stage were then obtained to confirm whether the publications met the eligibility criteria. At both the title and abstract and the full publication review stages, any discrepancies between reviewers were resolved through discussion or the intervention of a strategic advisor. Data from eligible studies were summarised in a narrative synthesis.

**Table 1:** SLR inclusion criteria

Criteria	Include	Exclude
Population	• Patients with gMG (including subtypes such as AChR + , MuSK + , seronegative, LPR4 + , early-onset, late-onset, refractory, or crisis MG as well as biological sex) • Mixed MG populations that exceed the prespecified proportion of gMG patients (> 80%)	• Patients with oMG • Mixed MG populations that are ≤ 80% gMG patients
Intervention and comparator(s)	No restriction	NA
Outcomes	• Patient burden, measured using: o Generic PROMs, e.g., EQ-5D (EQ-5D-3L, EQ-5D-5L), SF-36, WPAI, fatigue, treatment satisfaction, HADS, SF8, STAI, PTSD checklist, PHQ-9, FACIT fatigue scale, PGIS, PGIC o Disease-specific PROMs, e.g. MG-ADL, MG-QoL15 o Disability related to uncontrolled symptoms o Treatment-related comorbidities o Factors associated with increased impact (e.g. gender, age, income) • Patient experience/voice o Psychological impact o Fear o Lifestyle adaptations – home, work/occupation, hobbies, travels o Impact on family planning • Economic burden/resource use o Presenteeism/absenteeism o Out-of-pocket treatment costs o Hospital/ICU length of stay o Number of outpatient visits o Wider societal impact o Access to specialist care o Impact on family planning	NA
Study design	• Observational studies to include: o Epidemiological studies o Cohorts o Cross sectional studies o Patient surveys o Registries o Case series • Government/regulatory reports • Reports from other companies	• Studies conducted in a controlled, clinical setting • Single case studies/reports • PROM validation studies • Narrative/systematic reviews^a^
Geography	EMEA (data for mixed geographic region were of interest if at least one region of interest were included)	NA
Date of publication	No restriction for journal articles	Pre-2019 conference abstracts
Language of publication	English language publications or non-English language publications with an English abstract	NA

^a^Narrative/systematic reviews were excluded as they do not report novel data; however, the reference lists of review articles were hand searched to identify any additional eligible primary studies that were not identified in electronic database searches

Formal quality assessment using a validated checklist was not undertaken due to the anticipated heterogeneity in study design between relevant studies. However, key study characteristics that may impact the validity of the results (e.g. patient sample size, patient withdrawal and study perspective) were summarised to assist with establishing the robustness of the results reported in individual studies.

## Results

**Fig. 1:**
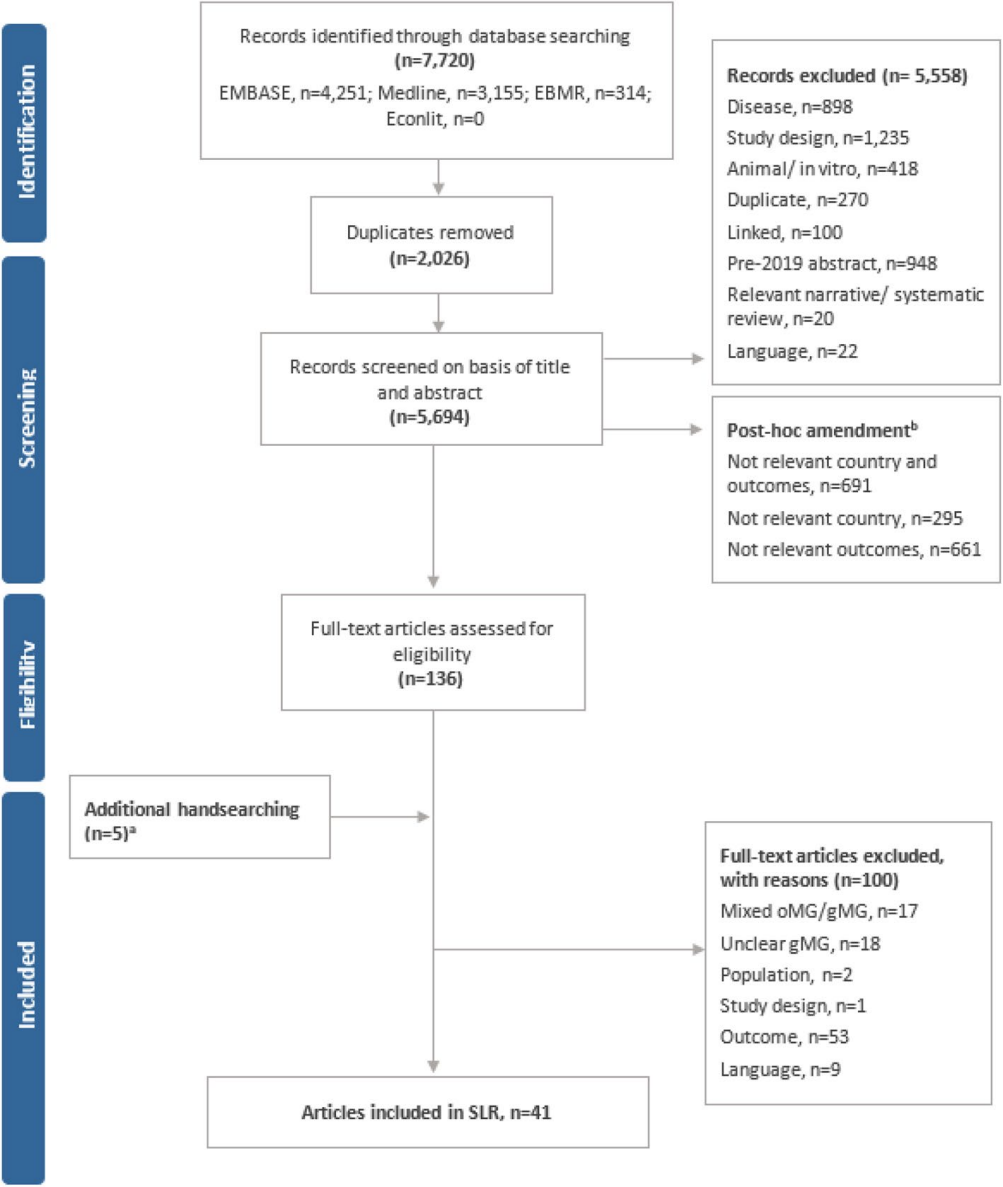
PRISMA diagram. **A** Including studies tagged on the basis of country and systematic reviews. **B** To ensure the most relevant data was being considered for inclusion, a post-hoc amendment to the protocol was included to exclude studies during title/abstract screening that did not indicate relevant outcome data

The process of study selection is documented in the PRISMA flow diagram (Fig. 1). The electronic database search identified a total of 7,720 articles. After the removal of 2,026 duplicates, 5,694 articles were screened by title and abstract. In total, 5,558 articles were excluded. The remaining 136 articles were deemed potentially relevant and subsequently screened based on the full publication. Hand searching of conference proceedings, additional sources, and reference lists of included studies yielded five additional relevant publications. Upon review of the full publications, a further 100 articles were excluded. This resulted in a total of 41 publications that met the inclusion criteria for the SLR. A list of the included studies and a summary of their key characteristics is provided in Table 2.

**Table 2:** Summary of included studies (*n* = 41)

Author (date)	Country	Study design	gMG data type (n/N)	gMG classification tool	PROMs
**Akkan Suzan 2022 **[1]	Turkey	Case–control	gMG subgroup data (30/53)	MGFA IIA	**Symptom-specific** (FAS, FIS, BDI, ESS)
**Alanazy 2019 **[3]	Saudi Arabia	Cross-sectional	gMG subgroup data (82/104)	Author reported	**Symptom-specific** (PHQ-9)
**Ambrogi 2012 **[4]	Italy	Retrospective cohort	All gMG (59/59)	MGFA III	**Generic** (SF-36)
**Andersen 2021 **[5]	Denmark	Cross-sectional	All gMG (100/100)	Author reported	**Generic** (EQ-5D-3L, EQ-5D-VAS, PASS) **Symptom-specific** (MFI-20, MDI) **Disease-specific** (MG-ADL, MG-QoL 15)
**Aysal 2013 **[6]	Turkey	Cross-sectional	gMG subgroup data (QoL by MG stage) and > 80% gMG (QoL by treatment) (36/42)	Osserman IIA-IIB	**Symptom-specific** (BDI, BAI, HAM-D, HAM-A)
**Bachmann 2008 **[7]	Germany	Prospective cohort	All gMG (106/106)	Osserman II-IV	**Generic** (EORTC QLQ)
**Baram 2021 **[8]	Iraq	Retrospective cohort	> 80% gMG with no subgroup data (44/48)	MGFA IIA-IVB	**Disease-specific** (MG-ADL)
**Bartel 1995 **[9]	South Africa	Case–control	> 80% gMG with no subgroup data (15/16)	Author reported	**Generic** (POMS) **Symptom-specific** (IPAT anxiety scale)
**Basta 2012 **[10]	Serbia	Cross-sectional	gMG subgroup data (120/230)	MGFA IIA-IIIB	**Generic** (SF-36)
**Birnbaum 2021 **[11]	France	Case–control	All gMG (33/33)	MGFA II-III	**Disease-specific** (MG-ADL, MG-QoL 15)
**Busch 1996 **[13]	Germany	Retrospective cohort	> 80% gMG with no subgroup data (61/65)	Modified Osserman II-IV	**Generic** (EORTC)
**Cioncoloni 2016 **[15]	Italy	Cross-sectional	gMG subgroup data (29/41)	MGFA IIA-IVB	**Disease-specific** (subjective patient evaluation [first item of the IMGQ])
**De Freitas Fregonezi 2006 **[16]	Spain	Prospective cohort	All gMG (20/20)	Osserman IIA-IIB	**Generic** (SF-36)
**De Lapiscina 2012 **[17]	Spain	Cross-sectional	gMG subgroup data (23/54)	Author reported	**Symptom-specific** (ESS, PSQI) **Disease-specific** (MG-QoL 15)
**Dewilde 2022 **[18]	Belgium, Canada, Germany, Italy, Japan, Spain, UK, USA	Prospective cohort	gMG subgroup data (NR/617)	MGFA II-IV	**Generic** (EQ-5D-5L)
**Happe 2004 **[23]	Austria	Case–control	> 80% gMG with no subgroup data (16/17)	Osserman II	**Generic** (QLI) **Symptom-specific** (ESS, SDS, SAS, PSQI, SSA)
**Hoffmann 2016 **[24]	Germany	Cross-sectional	gMG subgroup data (116/200)	MGFA II-IV	**Symptom-specific** (CFQ, HADS-D, HADS-A, ISI)
**Jastrzebska 2019 **[26]	Poland	Cross-sectional	> 80% gMG with no subgroup data (87/101)	MGFA	**Disease-specific** (MG-ADL)
**Jordan 2017 **[29]	Germany	Case–control	All gMG (33/33)	MGFA	**Generic** (10-point VAS) **Symptom-specific** (CES-D, PSQI, FSMC) **Disease-specific** (MGFS, MG-ADL, MG-QoL 15)
**Jordan 2017 **[28]					
**Kaukiainen 1977 **[30]	Finland	Cross-sectional	gMG subgroup data (169/181)	Oosterhuis	**None** (economic only)
**Kotan 2016 **[31]	Turkey	Cross-sectional	> 80% gMG with no subgroup data (43/52)	Author reported	**Generic** (PAIS-SR, MSPSS, PTGI, SF-36) **Symptom-specific** (HADS-D, HADS-A)
**Law 2021 **[32]	France, UK, USA	Retrospective cohort	All gMG (NR)	Author reported	**Patient lived experience**
**Lehnerer 2021 **[33]	Germany	Cross-sectional	gMG subgroup data (1,127/1,660)	Author reported	**Generic** (ESSI) **Symptom-specific** (HADS-D, HADS-A, CFQ) **Disease-specific** (MG-ADL, MG-QoL 15)
**Ohlraun 2015 **[36]	Germany	Cross-sectional	> 80% gMG with no subgroup data (642/791)	MGFA II-V	**Family planning**
**Onyekwulu 2010 **[37]	Nigeria	Retrospective cohort	All gMG (11/11)	Osserman IV	**None** (economic only)
**Padua 2001 **[38]	Italy	Prospective cohort	> 80% gMG with no subgroup data (44/46)	Osserman II-IV	**Generic** (SF-36)
**Peres 2017 **[40]	Portugal	Retrospective cohort	All gMG (6/6)	Author reported	**Generic** (EQ-5D) **Disease-specific** (MG-QoL 15)
**Raggi 2010 **[41]	Italy	Prospective cohort	gMG subgroup data (48/102)	MGFA II-IV	**Generic** (SF-36)
**Rodolico 2021 **[43]	Italy	Retrospective cohort	All gMG (15/15)	MGFA II-III	**Disease-specific** (MG-ADL)
**Roth 2002 **[44]	Switzerland	Retrospective cohort	All gMG (23/23)	Osserman II-IV, Oosterhuis	**Generic** (subjective patient evaluation)
**Ruckert 2003 **[45]	Germany	Retrospective cohort	All gMG (182/182)	Osserman II-III	**Disease-specific** (MG-ADL)
**Ruiter 2021 **[46]	Belgium	Cross-sectional	gMG subgroup data (NR/NR)	Positive scores on MG-ADL item(s) 5 and/or 6	**Symptom-specific** (CIS-f)
**Sabre 2017 **[47]	Estonia	Cross-sectional	> 80% gMG with no subgroup data (29/36)	Author reported	**Symptom-specific** (FSS)
**Sitek 2009 **[48]	Poland	Case–control	> 80% gMG with no subgroup data (29/33)	MGFA II-IV	**Symptom-specific** (BDI)
**Stankovic 2018 **[49]	Serbia	Prospective cohort	> 80% gMG with no subgroup data (70/73)	MGFA IIA-IV	**Generic** (MSPSS, AIS, SF-36) **Symptom-specific** (HAM-A, HAM-D)
**Stojanov 2019 **[50]	Serbia	Prospective cohort	gMG subgroup data (NR/70)	MGFA II-IV	**Generic** (SF-36) **Symptom-specific** (HAM-A, HAM-D) **Disease-specific** (MG-QoL 15r)
**Szczudlik 2020 **[51]	Poland	Cross-sectional	gMG subgroup data (228/339)	MGFA IIA-IVB	**Generic** (SF-36)
**Tascilar 2018 **[52]	Turkey	Case–control	All gMG (19/19)	MGFA II-III	**Symptom-specific** (ESS, PSQI, FSS, HAM-A, HAM-D) **Disease-specific** (MG-QoL 15)
**Thomsen 2021 **[54]	Denmark	Prospective cohort	> 80% gMG with no subgroup data (95/107)	MGFA II-IV	**Disease-specific** (MG-ADL, MG-QoL 15)
**Westerberg 2018 **[55]	Sweden	Cross-sectional	gMG subgroup data (31/40)	Author reported	**Disease-specific** (MG-QoL 15)

**Fig. 2:**
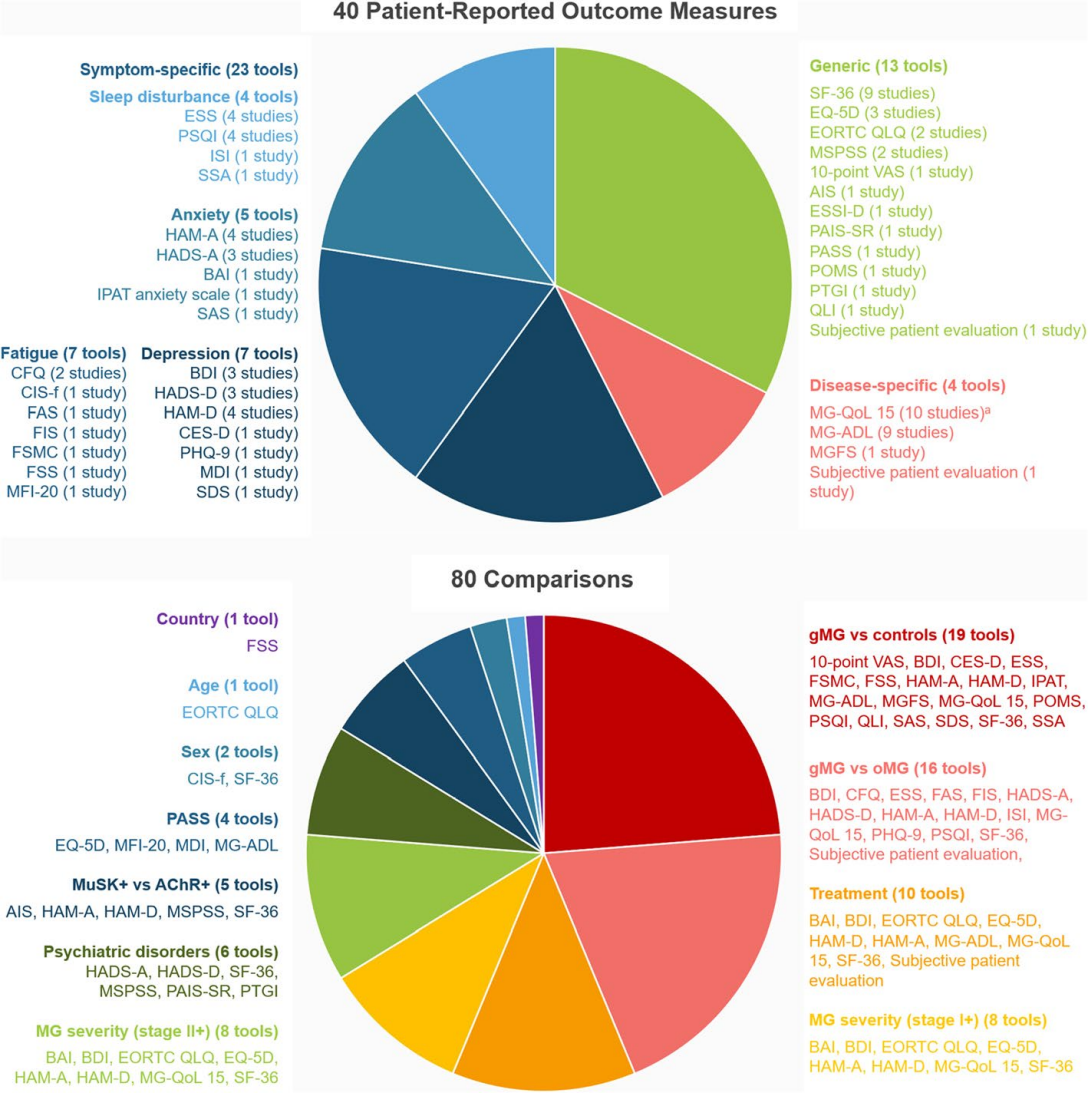
Visual summary of the heterogeneity in the PROMs and comparisons evaluated across the included studies. **A** One study used the revised version of the MG-QoL-15

Of the 41 included publications, 39 (representing 38 unique studies) reported on patient burden, consisting of formal patient-reported outcome measures (PROMs) (*n* = 37) [1, 3–11, 13, 15–18, 23, 24, 26, 28, 29, 31, 33, 38, 40, 41, 43–52, 54, 55] (Fig. 2), or alternative assessments of patient experience (*n* = 2) [32, 36]. The remaining two publications reported only on outcomes related to economic burden and are not the focus of this article [30, 37]. The majority of the patient burden studies were conducted in Europe (*n* = 32) [1, 4–7, 11, 13, 15–18, 23, 24, 26, 28, 29, 31–33, 36, 38, 40, 41, 43–48, 51, 52, 54, 55] with five studies conducted in Middle Eastern countries [3, 8, 10, 49, 50], and one study conducted in South Africa [9].

Overall, 12 patient burden studies recruited entirely gMG populations [4, 5, 7, 11, 16, 28, 29, 32, 40, 43–45, 52], and 15 studies recruited a mixed MG population but reported subgroup data for gMG [1, 3, 6, 10, 15, 17, 18, 24, 33, 41, 46, 47, 50, 51, 55]. In the remaining 11 studies a mixed MG population was reported with no gMG subgroup data [8, 9, 13, 23, 26, 31, 36, 38, 48, 49, 54]. However, the proportion of gMG patients in the population exceeded the pre-specified proportion of 80% in all 11 studies and data were therefore extracted for this combined population and considered equivalent to gMG. Only one study reported both gMG subgroup data and outcomes for an overall population that was > 80% gMG [6]. In this case data were extracted for the gMG subgroup where available, with the remaining outcomes extracted based on the full study population. The total sample size of the included studies (including non-gMG patients) ranged from 6–1,660 patients [33, 40], with approximately half of the studies including less than 50 patients with gMG [1, 3, 6, 8, 9, 11, 15–17, 23, 28, 29, 31, 38, 40, 41, 44, 47, 48, 52, 55]. An overview of trends identified in the extracted data is presented in Table 3, and summarised descriptively in the subsequent sections.

**Table 3:** Summary of trends identified in extracted data

Comparison	Generic QoL	Symptom-specific QoL	Disease-specific QoL
gMG patients vs healthy controls	**Significant difference (gMG worse):** Bartel 1995 (POMS) [9] De Freitas Fregonezi 2006 (SF-36) [16] Jordan 2017 (VAS) [28] **Numerical difference (significance not reported):** Padua 2001 (SF-36) [38]	**Significant difference (gMG worse):** Bartel 1995 (IPAT B-score) [9] Happe 2004 (SDS, SAS, PSQI, SSA) [23] Jordan 2017 (CES-D, PSQI, FSMC) [28, 29] Sitek 2009 (BDI) [48] Tascilar 2018 (PSQI, FSS, HAM-A, HAM-D) [52]	**Significant difference (gMG worse):** Jordan 2017 (MGFS, MG-ADL, MG-QoL 15) [28, 29]
	**No significant difference:** Happe 2004 (QLI) [23] Jordan 2017 (VAS cognitive) [29]	**No significant difference:** Bartel 1995 (IPAT A-score) [9] Happe 2004 (ESS) [23] Tascilar 2018 (ESS, adjusted PSQI, adjusted FSS) [52]	
gMG vs oMG	**Significant difference (gMG worse):** gMG vs oMG vs bMG: Stojanov 2019 (SF-36) [50] **Numerical difference (significance not reported):** gmG vs oMG: Cioncoloni 2016 (subjective measure) [15]	**Significant difference (gMG worse):** gMG vs oMG: Akkan Suzan 2022 (FIS total, FIS cognitive) [1] gMG vs oMG vs remission: Hoffmann 2016 (CFQ, ISI) [24] gMG vs oMG vs bMG: Stojanov 2019 (HAM-A) [50]	**Significant difference (gMG worse):** gMG vs oMG vs bMG: Stojanov 2019 (revised MG-QoL 15) [50] With vs without current gMG symptoms: Westerberg 2018 (MG-QoL 15) [55] **Numerical difference (significance not reported):** gMG vs oMG: De Lapiscina 2012 (MG-QoL 15) [17]
		**No significant difference:** Akkan Suzan 2022 (ESS, FIS physical, FIS social, FAS, BDI) [1] Alanazy 2019 (PHQ-9) [3] De Lapiscina 2012 (ESS, PSQI) [17] Hoffmann 2016 (HADS-D, HAS-A) [24] **Significant difference (oMG worse):** gMG vs oMG vs bMG: Stojanov 2019 (HAM-D) [50]	
MG severity (stage I +)	**Significant difference (higher stage worse):** MGFA: Basta 2012 (SF-36) [10] Osserman: Busch 1996 (modified EORTC QLQ) [13] MGFA: Dewilde 2022 (EQ-5D-5L) [18] Osserman: Padua 2001 (SF-36) [38] MGFA: Raggi 2010 (SF-36) [41] MGFA: Stojanov 2019 (SF-36) [50] MGFA: Szczudlik 2020 (SF-36) [51]	**Significant difference (higher stage worse):** Osserman: Aysal 2013 (BAI, HAM-D, HAM-A) [6] MGFA: Stojanov 2019 (HAM-A, HAM-D) [50]	**Significant difference (higher stage worse):** MGFA: Stojanov 2019 (revised MG-QoL 15) [50]
		**No significant difference:** Osserman: Aysal 2013 (BDI) [6]	
MG severity within gMG (stage II +)	**Significant difference (higher stage worse):** Osserman: Bachmann 2008 (EORTC QLQ) [7] MGFA: Szczudlik 2020 (SF-36) [51] **Numerical difference (significance not reported between gMG stages):** MGFA: Basta 2012 (SF-36) [10] Osserman: Busch 1996 (modified EORTC QLQ) [13] Osserman: De Freitas Fregonezi 2006 (SF-36) [16] MGFA: Dewilde 2022 (EQ-5D-5L) [18] Osserman: Padua 2001 (SF-36) [38] MGFA: Raggi 2010 (SF-36) [41] MGFA: Stojanov 2019 (SF-36) [50]	**Significant difference (higher stage worse):** Osserman: Aysal 2013 (BAI, HAM-D, HAM-A) [6] **Numerical difference (significance not reported between gMG stages):** MGFA: Stojanov 2019 (HAM-A, HAM-D) [50]	**Numerical difference (significance not reported between gMG stages):** MGFA: Stojanov 2019 (revised MG-QoL 15) [50]
		**No significant difference:** Osserman: Aysal 2013 (BDI) [6]	
Age	**Significant difference (older worse):** > 45 vs ≤ 45 years: Bachmann 2008 (EORTC QLQ) [7]	NR	NR
Sex	**No significant difference:** De Freitas Fregonezi 2006 (SF-36) [16]	**Significant difference (female worse):** Ruiter 2021 (CIS-f) [46]	NR
Country	NR	**Significant difference (Estonia worse):** Estonia vs Sweden, Sabre 2017 (FSS) [47]	NR
Impact of treatment	**Significant difference (improvement):** Thymectomy: Ambrogi 2012 (SF-36) [4] Thymectomy, failure vs response: Busch 1996 (EORTC QLQ) [13] **Numerical difference (significance not reported):** Rituximab: Peres 2017 (EQ-5D) [40] Thymectomy: Roth 2002 (subjective evaluation) [44]	**Significant difference (combination better):** Prednisolone alone vs prednisolone with azathioprine/pyridostigmine: Aysal 2013 (BAI, HAM-D, HAM-A total, HAM-A somatic) [6]	**Significant difference (improvement):** Thymectomy: Baram 2021 (MG-ADL) [8] Methotrexate: Rodolico 2021 (MG-ADL) [43] Standard care: Thomsen 2021 (MG-ADL, MG-QoL 15) [54] **Numerical difference (significance not reported):** Rituximab: Peres 2017 (MG-QoL 15) [40]
	**No significant difference:** Thymectomy, open vs minimally invasive: Bachmann 2008 (EORTC QLQ) [7] Thymectomised vs not thymectomised: Padua 2001 (SF-36) [38]	**No significant difference:** Prednisolone alone vs prednisolone and azathioprine/pyridostigmine: Aysal 2013 (BDI, HAM-A psychic) [6]	**No significant difference:** Thymectomy, tThx vs aThx vs sThx: Ruckert (MG-ADL) 2003 [45]
MuSK + MG vs AChR + MG	**Significant difference (AChR worse):** Stankovic 2018 (MSPSS, SF-36) [49]	**No significant difference:** Stankovic 2018 (HAM-A, HAM-D) [49]	NR
	**No significant difference:** Stankovic 2018 (AIS) [49]		
PASS-postive vs PASS-negative	**Significant difference (PASS-negative worse):** Andersen 2021 (EQ-5D-3L, EQ-5D-VAS) [5]	**Significant difference (PASS-negative worse):** Andersen 2021 (MFI-20, MDI) [5]	**Significant difference (PASS-negative worse):** Andersen 2021 (MG-ADL) [5]
With vs without psychiatric disorders	**Significant difference (psychiatric disorders worse):** Kotan 2016 (SF-36, MSPSS, PAIS-SR) [31]	**Significant difference (psychiatric disorders worse):** Kotan 2016 (HADS-A, HADS-D) [31]	NR
	**No significant difference:** Kotan 2016 (PTGI) [31]		

### Generic PROMs

In total, 20 publications (representing 19 unique studies) reported the results of non-symptom-specific generic QoL measures in patients with gMG (Fig. 2). The most common measures were the 36-Item Short Form Survey (SF-36) (*n* = 9) [4, 10, 16, 31, 38, 41, 49–51], EuroQoL Five Dimension Questionnaire 3 Levels (EQ-5D-3L) or 5 Levels (EQ-5D-5L) (*n* = 3) [5, 18, 40], and European Organisation for Research and Treatment of Cancer Quality of Life Questionnaire (EORTC QLQ) (*n* = 2) [7, 13]. Other PROMs that were not symptom- or MG-specific included measures related to social support [31, 33, 49]; acceptance of illness [31, 49]; post-traumatic growth [31]; patient mood [9]; and general QoL [23, 28, 29, 44].

#### Impact of gMG on QoL

Three of the studies investigating non-symptom-specific generic PROMs reported significantly lower scores in patients with gMG compared with healthy controls [9, 16, 28, 29]. SF-36 scores were found to be significantly lower in gMG patients compared with healthy reference values for physical functioning, role limitation due to physical problems, and general health perceptions [16]. A study assessing patients using the Profile of Mood States (POMS) found that gMG patients had significantly higher scores for tension, anger, fatigue and confusion [9]. Another study found that patients’ perceived level of physical and cognitive performance on a 10-point visual analogue scale (VAS) was significantly lower in gMG patients versus controls [28, 29]. In contrast to these findings, one study reported that the difference in quality of life index (QLI) score between gMG patients and healthy controls was narrowly non-significant [23].

#### Severity of gMG

A number of studies assessed the relationship between general QoL measures and MG classification, as assessed by the Osserman and Myasthenia Gravis Foundation of America (MGFA) classification systems, which range from stage I (oMG) to stage IV/V, respectively [27, 53]. The majority of these studies did not provide statistical analysis of the impact of increasing severity within gMG (stage II +); however, there was typically a trend for worsening QoL between stage II and higher stages [7, 10, 16, 18, 41, 51]. One study reported significantly lower EORTC QLQ scores for social, cognitive, emotional and vegetative scales for patients in stage III/IV versus stage II [7], while a second study reported a significantly lower SF-36 physical functioning score for patients in MGFA stage III or more compared with stage II [51]. A final study found that SF-36 scores differed significantly between patients with gMG, oMG and bulbar MG (bMG), which impacts the jaw and throat muscles [50]. QoL scores were highest in patients with oMG and lowest in patients with bMG [50].

#### Treatment

Six of the included studies evaluated non-symptom-specific general QoL in patients after receiving specific treatments for gMG, including thymectomy (*n* = 5) [4, 7, 13, 38, 44] and rituximab (*n* = 1) [40]. The rituximab study found a positive tendency towards an improvement in EQ-5D-3L overall score and VAS following treatment [40]. Findings related to the impact of thymectomy on QoL were inconclusive, with one study reporting a significant improvement in SF-36 over time following thymectomy up to a maximum of 10 years [4], while a second study reported no difference in SF-36 between patients with and without thymectomies [38]. One study found no significant differences in EORTC QLQ scores between gMG patients undergoing open thymectomy versus minimally invasive thoracoscopic thymectomy [7]. A further study reported improvements in EORTC QLQ score following thymectomy with a mean follow-up of over 7 years [13]. The final study reported patient’s subjective evaluation of their QoL following thymectomy, finding that the majority of patients considered themselves to be in good or very good condition after an average of 13 years of follow-up [44].

#### Other factors affecting QoL

One study evaluated differences in gMG patients’ quality of life based on their Patient Acceptable Symptom State (PASS), a single-item assessment in which patients indicate whether they are satisfied with their current symptom state (PASS-positive) or dissatisfied with their current symptom state (PASS-negative) [5]. PASS-negative gMG patients had significantly lower EQ-5D-3L and EQ-5D-VAS scores than PASS-positive gMG patients [5].

Three studies reported on PROMs assessing social support, including the Multidimensional Scale of Perceived Social Support (MSPSS) (*n* = 2) [31, 49], and the ENRICHD Social Support Inventory (ESSI) (*n* = 1) [33]. One study found that MSPSS score correlated with total SF-36 score, and that MSPSS was higher in MG patients with autoantibodies against muscle-specific tyrosine kinase (MuSK + MG) than in MG patients with autoantibodies to acetylcholine receptor (AChR + MG) [49]. This may be due to the more severe symptoms associated with MuSK + MG versus AChR + MG, resulting in greater support from friends or family members [49]. Overall, SF-36 score was better in MuSK + MG patients, particularly in mental domains, despite these patients tending to have a more severe form of the disease [49]. The second MSPSS study assessed QoL in patients with gMG with and without a psychiatric diagnosis, finding that MSPSS scores were significantly higher in patients without a psychiatric diagnosis, and that MSPSS score correlated with SF-36 general health score; with patients with a psychiatric diagnosis having worse SF-36 scores than patients without a psychiatric diagnosis [31].

Two studies reported PROMs related to patients’ acceptance of or adjustment to living with gMG: the Psychosocial Adjustment to Illness Scale – Self Report (PAIS-SR) [31], and the Acceptance of Illness Scale (AIS) [49]. The AIS study found that patients’ scores did not differ between MuSK + MG and AChR + MG [49]. The second study found that PAIS-SR score was significantly lower in gMG patients without a psychiatric diagnosis than those with a psychiatric diagnosis, and that PAIS-SR score correlated with Hospital Anxiety and Depression Scale (HADS) scores [31]. In the same study, patients were also asked to complete the Post-Traumatic Growth Inventory (PTGI) assessing whether they had experienced positive changes after trauma; no significant difference in PTGI scores was identified between the two groups [31].

### Symptom-specific PROMS

In total, 18 publications (representing 17 unique studies) reported on symptom-specific PROMs (Fig. 2), including measures of depression (*n* = 14) [1, 3, 5, 6, 23, 24, 28, 29, 31, 33, 48–50, 52], anxiety (*n* = 9) [6, 9, 23, 24, 31, 33, 49, 50, 52], fatigue (*n* = 9) [1, 5, 24, 28, 29, 33, 46, 47, 52], and sleep disturbance (*n* = 7) [1, 17, 23, 24, 28, 29, 52].

#### Depression

The most frequently reported depression-related PROMs were the Hamilton Depression Rating Scale (HAM-D) (*n* = 4), [6, 49, 50, 52], Beck's Depression Inventory (BDI) (*n* = 3) [1, 6, 48], and HADS depression subscale (*n* = 3) [24, 31, 33], with the remaining measures reported in one study each: the Center for Epidemiologic Studies Depression Scale (CES-D) [28, 29], Major Depression Inventory (MDI) [5], Patient Health Questionnaire (PHQ)-9 [3], and the Zung Self-Rating Depression Scale (SDS) [23]. Four studies reported significantly higher scores for gMG patients compared with healthy controls on the HAM-D [52], BDI [48], CES-D, and SDS [23] scales. Findings on MG severity were mixed, one study found that HAM-D score increased with more severe Osserman stage, while there was no significant difference in BDI across stages [6]. A further study found that HAM-D score varied significantly between oMG, gMG and bMG, with oMG patients having the worst scores [50]. In contrast, three studies reported no significant differences between gMG and oMG on the HADS-D [24], BDI [1] and PHQ-9 [3] scales. Other findings included a significantly higher HADS-D score in gMG patients with psychiatric disorders versus gMG patients without such disorders [31], and similar HAM-D scores in patients with MuSK + MG and AChR + MG [49].

#### Anxiety

The most frequently reported anxiety-related PROMs were the Hamilton Anxiety Rating Scale (HAM-A) (*n* = 4) [6, 49, 50, 52] and HADS-A (*n* = 3) [24, 31, 33], with the remaining measures reported in one study each: Beck Anxiety Inventory (BAI) [6], Institute for Personality & Ability Testing (IPAT) Anxiety Scale [9], and the Zung Self-Rating Anxiety Scale (SAS) [23]. Three studies found significantly higher anxiety scores in patients with gMG versus healthy controls, using the IPAT B-score [9], SAS [23], and HAM-A [52] scales. Two studies reported higher HAM-A scores at higher MGFA [50] and Osserman stages [6], and one study found that HAM-A was higher in patients with gMG versus oMG [50]. In contrast, a further study found no significant difference in HADS-A score between patients with gMG and oMG [24]. Other findings included a significantly higher HADS-A score in gMG patients with psychiatric disorders versus gMG patients without such disorders [31], and similar HAM-A scores in patients with MuSK + MG and AChR + MG [49].

#### Fatigue

The measures used to assess fatigue were highly variable. The Chalder Fatigue Questionnaire (CFQ) [24, 33] and Fatigue Severity Scale (FSS) [47, 52] were each reported in two studies, with the remaining measures reported in one study each: Checklist Individual Strength fatigue (CIS-f) [46], Fatigue Assessment Scale (FAS) [1], Fatigue Impact Scale (FIS) [1], Fatigue Scale for Motor and Cognitive Functions (FSMC) [28, 29], Multidimensional Fatigue Inventory (MFI)-20 [5]. Two studies assessed fatigue in patients with gMG versus healthy controls, finding that fatigue scores were significantly higher for gMG patients on the FSS [52], and FSMC scales (23,24). Two further studies found a significant difference between gMG and oMg patients, with gMG patients having higher scores on the CFQ [24] and FIS scales [1]. Other findings included significantly higher CIS-f scores in women with gMG than in men with gMG [46], and significantly higher MFI-20 scores in PASS-negative gMG patients than in PASS-positive gMG patients [5].

#### Sleep disturbance

The most frequently reported sleep-related PROMs were the Epworth Sleepiness Scale (ESS) [1, 17, 23, 52] and Pittsburgh Sleep Quality Index (PSQI) [17, 23, 28, 29, 52], reported in four studies each, with the remaining measures reported in one study each: Insomnia Severity Index (ISI) [24], Self-Rating Questionnaire for Sleep and Awakening Quality (SSA) [23]. Sleep-related findings in gMG patients were mixed. Two studies found no difference in ESS total score between gMG patients and healthy controls [23, 52]; whereas, PSQI score was higher in gMG patients than controls in three studies [23, 28, 29, 52], as was SSA score in one study [23]. A further study reported that ISI score was higher in patients with gMG versus oMG [24], with a final study reporting a significant relationship between QoL and subjective sleep duration, as well as finding that sleep disorders were more prevalent in the gMG population than in healthy controls [52].

## Disease-specific PROMs

In total, 16 publications (representing 15 unique studies) reported disease-specific PROMs in patients with gMG (Fig. 2). The most common were Myasthenia Gravis Quality of Life (MG QoL)-15 (*n* = 10 studies [5, 11, 17, 28, 29, 33, 40, 50, 52, 54, 55], one of which employed the revised version, MG QoL-15r [50]), and Myasthenia Gravis Activities of Daily Living (MG-ADL) (*n* = 9) [5, 8, 11, 26, 28, 29, 33, 43, 45, 54]. Two other measures were reported in one study each: the Italian Myasthenia Gravis Questionnaire (IMGQ) [15] and the Myasthenia Gravis Fatigue Scale (MGFS) [15]. One study assessed MGFS, MG-ADL and MG-QoL 15 in patients with gMG versus healthy controls, finding significantly worse values in gMG patients [28, 29]. Two studies reported significantly lower scores in patients with gMG compared with oMG on the MG QoL-15 [55] and MG QoL-15r [50], whereas one study reported a numerically lower MG QoL-15 score in patients with gMG vs oMG or patients in remission [17]. A final study reported an increase in MG QoL-15r score with increasing MGFA stage [50].

Five studies reported on disease-specific QoL in patients before and after receiving various treatments for MG, including thymectomy (*n* = 3) [8, 26, 45], rituximab (*n* = 1) [40], methotrexate (*n* = 1) [43], and standard care (*n* = 1) [54]. Four of these studies reported an improvement in disease-specific QoL following treatment [8, 40, 43, 54], one study found no difference in MG-ADL score across different thymectomy approaches [45], and the remaining study assessed MG-ADL in patients who had undergone thymectomy but the researchers were unable to evaluate the change in QoL as the data were incomplete [26]. Other findings included a significantly higher MG-ADL score in PASS-negative compared with PASS-positive patients [5].

### Patient experience

Two publications did not use formal tools to assess quality of life, instead conducting qualitative evaluations of patient experience [32, 36]. One publication focused on family planning decision-making in women with gMG, finding that gMG influenced family planning in the majority of patients [36]. The second publication reported summary statements describing the lived experience of patients with gMG, which were generated in an analysis led by a panel of patient advocates and informed by patient insights [32]. Five key themes were identified encompassing fluctuating and unpredictable symptoms; trade-offs in all aspects of life; treatment inertia; disconnection from healthcare professionals; and feelings of anxiety, frustration, guilt, anger, loneliness and depression [32].

## Discussion

The objective of this systematic review was to identify and summarise the existing body of evidence for patient burden in MG, with a specific focus on patients experiencing generalised symptoms (gMG) in Europe, the Middle East and Africa. A total of 38 unique studies were identified as relevant for inclusion, encompassing 36 studies reporting the results of general, symptom-specific or disease-specific PROMs. Many of the included studies reported a substantial impact of gMG on patient QoL, with this impact increasing with increasing MG severity. This finding is in line with a recent paper showing that MGFA grade is a strong predictor of all aspects of health-related quality of life (HRQoL) in MG patients [19]. A systematic review of the humanistic burden of MG (Gelinas 2022) also drew similar conclusions, finding that patients with MG experience worse HRQoL than the general population [22]. The Gelinas 2022 SLR covered a broader data set than the present review; not being limited in geography, study design, and MG subtype. Our review also includes more recent data and draws attention to some key data gaps regarding patient burden in gMG. Furthermore, the majority of the studies identified in the review were conducted in Europe, illustrating that further studies from a broader range of countries are required to provide greater insight into the patient experience of gMG in the EMEA region.

Our review was conducted according to robust methodology, and a comprehensive data-set was obtained; however, there was substantial variation in sample sizes, patient populations and study design across the included studies. A total of 40 different tools were used across 38 studies, with a high level of heterogeneity in the comparisons analysed (Fig. 2). Some PROMs were only reported in a limited number of studies, and the differences in the tools limit our ability to summarise and compare across studies. There is therefore a need for further representative and well-powered studies in large cohorts administering consistent, validated questionnaires.

Our review also included searches for data relating to the economic burden of patients with gMG. Substantial data gaps were identified, with measures of economic burden of gMG primarily limited to impact of MG on work capability and healthcare resource use outcomes, such as hospitalisations or length of hospital stay [4, 5, 7, 8, 26, 30, 31, 36, 37, 43, 44]. Direct economic data were limited to a single cost-utility analysis, which reported reduced overall healthcare costs in six patients with gMG treated with rituximab [40]. The lack of available comparative data and the heterogeneity of the reported outcomes make it difficult to draw any conclusions regarding the economic burden of gMG, and these data are therefore not presented in this article.

In contrast to this uncertainty, the patient-led analysis of MG patient burden identified in the review clearly describes the impact of MG on QoL and emphasised the need for greater understanding of the reality of living with MG [32]. A limitation of this study is the geographical restriction to the EMEA region, which may reduce the generalisability of the findings. However, a recent study of gMG patient experience in the US found that patients report similar difficulties, including unpredictable symptoms that impact many aspects of life including social functioning, work capacity and finances [25]. Only two studies identified in this review assessed qualitative aspects of gMG patient burden [32, 36]. This limited focus on qualitative assessments of patient burden versus formal PROMs points to the need for further analyses in this particular area to better reflect patient’s lived experience of MG.

## Conclusions

Despite the limitations of the published literature, the patient burden of gMG remains clear, with this review identifying a range of studies that report a substantial impact of gMG on patient QoL. Key findings from the analysis of patient lived experience included concerns around treatment-inertia and undertreatment of MG, as well as a disconnect between patients and healthcare professionals in both the perception of disease burden and treatment goals [32]. This review therefore emphasises the importance of considering patient QoL when developing treatment and management plans for patients with gMG, thus ensuring that optimal support is provided to these patients.

### Supplementary Information

**Additional file 1. **

## Data Availability

All MG-related data extraction tables generated during this study are included in this published article or its Supplementary information files.

## Abbreviations

AIS: Acceptance of Illness Scale
AChR: Acetylcholine receptor
BAI: Beck Anxiety Inventory
BDI: Beck's Depression Inventory
bMG: Bulbar myasthenia gravis
CES-D: Center for Epidemiologic Studies Depression Scale
CFQ: Chalder Fatigue Questionnaire
CIS-f: Checklist Individual Strength fatigue
EBMR: Evidence-Based Medicine Reviews
EMEA: Europe, the Middle East and Africa
ENRICHD: Enhancing Recovery in Coronary Heart Disease
EORTC QLQ: European Organisation for Research and Treatment of Cancer Quality of Life Questionnaire
EQ-5D: EuroQoL Five Dimension Questionnaire
EQ-5D-3L: EuroQoL Five Dimension Questionnaire 3 Levels
EQ-5D-5L: EuroQoL Five Dimension Questionnaire 5 Levels
ESS: Epworth Sleepiness Scale
ESSI: ENRICHD Social Support Inventory
FACIT: Functional Assessment of Chronic Illness Therapy
FAS: Fatigue Assessment Scale
FcRn: Neonatal Fc receptor
FIS: Fatigue Impact Scale
FSMC: Fatigue Scale for Motor and Cognitive Functions
gMG: Generalised myasthenia gravis
HADS-A: Hospital Anxiety and Depression Scale Anxiety
HADS-D: Hospital Anxiety and Depression Scale Depression
HAM-A: Hamilton Anxiety Rating Scale
HAM-D: Hamilton Depression Rating Scale
HRQoL: Health-related quality of life
ICU: Intensive care unit
IMGQ: Italian Myasthenia Gravis Questionnaire
IPAT: Institute for Personality & Ability Testing
ISI: Insomnia Severity Index
LPR: Low density lipoprotein receptor-related protein
MDI: Major Depression Inventory
MFI: Multidimensional Fatigue Inventory
MG: Myasthenia gravis
MG-ADL: Myasthenia Gravis Activities of Daily Living
MGFA: Myasthenia Gravis Foundation of America
MGFS: Myasthenia Gravis Fatigue Scale
MG-QoL: Myasthenia Gravis Quality of Life
MSPSS: Multidimensional Scale of Perceived Social Support
MuSK: Muscle-specific tyrosine kinase
NA: Not applicable
oMG: Ocular myasthenia gravis
PAIS-SR: Psychosocial Adjustment to Illness Scale – Self Report
PASS: Patient Acceptable Symptom State
PGIC: Patients' Global Impression of Change
PGIS: Patient Global Impression of Symptom Severity
PHQ: Patient Health Questionnaire
POMS: Profile of Mood States
PRISMA: Preferred Reporting Items for Systematic Reviews and Meta-Analyses
PROM: Patient-reported outcome measure
PSQI: Pittsburgh Sleep Quality Index
PTGI: Post-Traumatic Growth Inventory
PTSD: Post-traumatic stress disorder
QLI: Quality of life index
QoL: Quality of life
SAS: Self-Rating Anxiety Scale
SDS: Self-Rating Depression Scale
SLR: Systematic literature review
SF8: Short Form-8 Health Survey
SF-36: 36-Item Short Form Survey
STAI: State-Trait Anxiety Inventory
SSA: Self-Rating Questionnaire for Sleep and Awakening Quality
VAS: Visual analogue scale
WPAI: Work productivity and activity impairment
