# Supplementary material for: Systematic review of the patient burden of generalised myasthenia gravis in Europe, the Middle East, and Africa
Source: BMC Neurol. 2024 Feb 10;24:61. doi: 10.1186/s12883-024-03553-y (PMC10858594; doi:10.1186/s12883-024-03553-y)
Supplement: Supplementary file 1 — Additional file 1. [file 12883_2024_3553_MOESM1_ESM.docx]

Supplementary Table 1: Embase Search Strategy

Embase (Ovid): 1974 to 2022 March 28: searched 29.3.2022

| # | Searches | Results |
| --- | --- | --- |
| 1 | *myasthenia gravis/ | 15315 |
| 2 | Myasthenia gravis.ti,ab. | 19056 |
| 3 | 1 or 2 | 21051 |
| 4 | Clinical study/ | 157657 |
| 5 | Case control study/ | 186033 |
| 6 | Family study/ | 25402 |
| 7 | Longitudinal study/ | 170020 |
| 8 | Retrospective study/ | 1222321 |
| 9 | Prospective study/ | 755679 |
| 10 | Randomized controlled trials/ | 223333 |
| 11 | 9 not 10 | 746923 |
| 12 | Cohort analysis/ | 823584 |
| 13 | (Cohort adj (study or studies)).mp. | 393674 |
| 14 | (Case control adj (study or studies)).tw. | 152629 |
| 15 | (follow up adj (study or studies)).tw. | 68798 |
| 16 | (observational adj (study or studies)).tw. | 212976 |
| 17 | (epidemiologic$ adj (study or studies)).tw. | 115161 |
| 18 | (cross sectional adj (study or studies)).tw. | 282630 |
| 19 | or/4-8,11-18 | 3367065 |
| 20 | ((real adj world) or RWE).ti,ab. | 101755 |
| 21 | (registr$ or register$ or database$).ti,ab. | 1405490 |
| 22 | health survey/ | 211324 |
| 23 | exp interview/ | 326187 |
| 24 | questionnaire/ or open ended questionnaire/ or structured questionnaire/ | 823478 |
| 25 | (survey$ or interview$ or questionnaire$).ti,ab. | 1978761 |
| 26 | (patient$ adj3 (burden or experience$ or voice)).ti,ab. | 263386 |
| 27 | or/20-26 | 3740978 |
| 28 | socioeconomics/ | 151571 |
| 29 | exp Quality of Life/ | 573510 |
| 30 | quality of life.ti,kw. | 155435 |
| 31 | ((instrument or instruments) adj3 quality of life).ab. | 5092 |
| 32 | Quality-Adjusted Life Year/ | 31127 |
| 33 | quality adjusted life.ti,ab,kw. | 23279 |
| 34 | (qaly* or qald* or qale* or qtime* or life year or life years).ti,ab,kw. | 39035 |
| 35 | disability adjusted life.ti,ab,kw. | 5150 |
| 36 | daly*.ti,ab,kw. | 5068 |
| 37 | (sf36 or sf 36 or short form 36 or shortform 36 or short form36 or shortform36 or sf thirtysix or sfthirtysix or sfthirty six or sf thirty six or shortform thirtysix or shortform thirty six or short form thirtysix or short form thirty six).ti,ab,kw. | 46347 |
| 38 | (sf6 or sf 6 or short form 6 or shortform 6 or sf six or sfsix or shortform six or short form six or shortform6 or short form6).ti,ab,kw. | 2702 |
| 39 | (sf8 or sf 8 or sf eight or sfeight or shortform 8 or shortform 8 or shortform8 or short form8 or shortform eight or short form eight).ti,ab,kw. | 951 |
| 40 | (sf12 or sf 12 or short form 12 or shortform 12 or short form12 or shortform12 or sf twelve or sftwelve or shortform twelve or short form twelve).ti,ab,kw. | 10994 |
| 41 | (sf16 or sf 16 or short form 16 or shortform 16 or short form16 or shortform16 or sf sixteen or sfsixteen or shortform sixteen or short form sixteen).ti,ab,kw. | 64 |
| 42 | (sf20 or sf 20 or short form 20 or shortform 20 or short form20 or shortform20 or sf twenty or sftwenty or shortform twenty or short form twenty).ti,ab,kw. | 489 |
| 43 | (hql or hqol or h qol or hrqol or hr qol).ti,ab,kw. | 34206 |
| 44 | (hye or hyes).ti,ab,kw. | 151 |
| 45 | (health* adj2 year* adj2 equivalent*).ti,ab,kw. | 52 |
| 46 | (pqol or qls).ti,ab,kw. | 695 |
| 47 | (quality of wellbeing or quality of well being or index of wellbeing or index of well being or qwb).ti,ab,kw. | 812 |
| 48 | (eq or euroqol or euro qol or eq5d or eq 5d or euroqual or euro qual).ti,ab,kw. | 32786 |
| 49 | (MG-ADL or MG-QoL15 or CFQ-11 or ESSI-D or HADS-D).ti,ab,kw. | 1802 |
| 50 | or/28-49 | 780744 |
| 51 | exp productivity/ | 44536 |
| 52 | human capital.mp. | 2995 |
| 53 | friction cost*.mp. | 217 |
| 54 | ((societ* or indirect) adj3 (cost or burden)).mp. [mp=title, abstract, heading word, drug trade name, original title, device manufacturer, drug manufacturer, device trade name, keyword heading word, floating subheading word, candidate term word] | 10215 |
| 55 | exp medical leave/ | 7796 |
| 56 | time off.mp. | 3279 |
| 57 | sick leave.mp. | 7593 |
| 58 | absent*.mp. | 231433 |
| 59 | exp absenteeism/ | 18669 |
| 60 | exp presenteeism/ | 1869 |
| 61 | exp caregiver burden/ or exp caregiver/ or exp caregiver support/ | 106474 |
| 62 | Caregiver*.mp. [mp=title, abstract, heading word, drug trade name, original title, device manufacturer, drug manufacturer, device trade name, keyword heading word, floating subheading word, candidate term word] | 138775 |
| 63 | or/51-62 | 430064 |
| 64 | 19 or 27 or 50 or 63 | 6880546 |
| 65 | 3 and 64 | 4251 |

Supplementary Table 2: Additional Sources

| Category | Sources |
| --- | --- |
| Reference lists | - Studies identified for inclusion in the review - Narrative/systematic reviews identified in electronic database searches |
| Conference proceedings (post-2019) | Hand searched or indexed in Embase:   - American Academy of Neurology (AAN) (<https://www.aan.com/>) – 2019, 2020, 2021 - American Association of Neuromuscular & Electrodiagnostic Medicine (AANEM) (<https://www.aanem.org/Home>) – 2019, 2020, 2021 - European Academy of Neurology (EAN) (<https://www.ean.org/>) – 2019, 2020, 2021 - European Conference on Rare Diseases & Orphan Products (ECRD) (<https://www.rare-diseases.eu/>) - 2020 - International Congress on Neuromuscular Diseases (ICNMD) (<https://icnmd.org/>) – 2020 (postponed to 2021) - International Society of Neuro Immunology (ISNI) (<https://www.isniweb.org/>) – 2020 (postponed to 2021) - International Society for Pharmacoeconomics and Outcomes Research (ISPOR) (<https://www.ispor.org/>) – 2019, 2020, 2021, 2022 - Muscular Dystrophy Association Clinical & Scientific Conference (MDA) (<https://mdaconference.org/>) – 2020, 2021 - Myasthenia Gravis Foundation of America (MGFA) (<https://myasthenia.org/>) – 2021 - Muscle Study Group Scientific Meeting (MSG) (<https://musclestudygroup.org/>) – 2019, 2020   The following conferences were also identified as relevant to the population of interest; however, abstracts could not be accessed:   - European Neurology Congress (<https://www.ean.org/>) - European Paediatric Neurology Society Congress (EPNS) (<https://www.epns.info/>) – 2019 - Muscular Dystrophy Association Clinical & Scientific Conference (MDA) (<https://mdaconference.org/>) – 2019 - Muscle Study Group Scientific Meeting (MSG) (<https://musclestudygroup.org/>) – 2021 |
| HTA global bodies | International Network of Agencies for Health Technology Assessment (INAHTA) website (<http://www.inahta.org/>) |
| Advocacy group websites | - Orpha Net (<https://www.orpha.net/consor/cgi-bin/SupportGroup.php?lng=EN>) - EURORDIS (<https://www.eurordis.org/>) - AFM Telethon (<https://www.myasthenie.fr/>) - AMES (<https://miastenia.ong/>) - AIM (<https://www.miastenia.it/>) - ASEM (<https://www.asem-esp.org/>) - DGM (<https://www.dgm.org/>) - DMG (<https://dmg-online.de/>) - Les Amis (<https://www.myasthenie.fr/>) - Liga MG (<https://www.ligamg.be/>) - MD UK (<https://www.musculardystrophyuk.org/>) - MyAware (<https://www.myaware.org/>) - Neuro (<https://www.asem-esp.org/>) |
| Utility databases | University of Sheffield ScHARRHUD (<http://www.scharrhud.org/>) |
| Google Scholar | NA |

Supplementary Table 3: Summary and definitions of PROMs used in the studies included in the systematic review

| PROM | Description of scale | Scale range | High score = high QoL | Number of studies (references) |
| --- | --- | --- | --- | --- |
| **General PROMs** | | | | |
| **SF-36** | A 36-item tool covering 8 health domains: physical functioning, social functioning, physical role, emotional role, vitality, body pain, mental health, and general health perception. Scored on varying scales by item (score: NA) | 0-100 (per domain) | ✓ | **9 studies** (1-9) |
| **EQ-5D** | A generic classification system for measuring health-related QoL, compromising the five dimensions each with three questions; mobility, self-care, usual activities, pain/discomfort, and depression/anxiety | 0-1 | ✓ | **3 studies** (10-12) |
| **EORTC QLQ** | A core of 30 generally applicable, unspecific items scored on a 4-point scale; a functional domain (items 1-5), a working ability domain (items 6 and 7), a general symptom domain (items 8-19), scales on cognitive (items 20 and 25), emotional (items 21-24), and social (items 26 and 27) functioning, a financial strain domain (item 28) and a global QoL domain (items 29 and 30) | 0-100 (per domain) | ✓ | **2 studies** (13, 14) |
| **MSPSS** | A 12-item tool to determine social support including three different types of support (support by partner, friends, and family members) scored on a seven-point scale (score: 1-7) | 12-84 | ✓ | **2 studies** (4, 6) |
| **10-point VAS** | A 10-point scale of self-perceived level of physical and cognitive performance before and after testing | 0-10 | ✓ | **1 study** (15, 16) |
| **AIS** | Eight dichotomous statements regarding acceptance of illness | Dichotomous | NA | **1 study** (6) |
| **ESSI-D** | A seven-item tool used to measure social support. The tool is a mixture of dichotomous and scale-based questions (score: NA) | 5-25 | ✓ | **1 study** (17) |
| **PAIS-SR** | A 46-item, multiple domain, semi-structured interview to assess the quality of a patient’s psychosocial adjustment to a current medical illness or the sequelae of a previous illness scored on a four-point scale (score: 0-3) | NR | 🗶 | **1 study** (4) |
| **PASS** | One dichotomous yes/no question; “Considering all the ways you are affected by Myasthenia gravis, if you had to stay in your current state for the next months, would you say that you are satisfied with your current disease state?” | Dichotomous | NA | **1 study** (10) |
| **POMS (adapted)** | A 25-item tool used to assess mood (tension, depression, anger, fatigue and confusion) over the preceding week (scoring NR) | NR | 🗶 | **1 study** (18) |
| **PTGI** | A 21-item tool to assess positive changes after traumatic experiences scored on a six-point scale (score: 0-5) | NR | 🗶 | **1 study** (4) |
| **QLI** | NR | NR | NR | **1 study** (19) |
| **Subjective patient evaluation** | Patients were asked whether they considered themselves to either be in very good condition, good condition, better than before surgery, did not observe any change, or felt worse than before surgery. | NA | NA | **1 study** (20) |
| **Anxiety PROMs** | | | | |
| **BAI** | A 21-item tool each defining an anxiety symptom scored on a four-point scale (score: 0-3) | 0-63 | 🗶 | **1 study** (21) |
| **HADS-A** | A seven-item tool developed to assess psychological distress in non-psychiatric patients scored on a four-point scale (score: 0-3) | 0-21 | 🗶 | **3 studies** (4, 17, 22) |
| **HAM-A** | A 14-item tool for evaluating psychic and somatic anxiety scored on a five-point scale (score: 0-4) | 0-56 | 🗶 | **4 studies** (6, 7, 21, 23) |
| **IPAT anxiety scale** | A 40-item tool distributed among five anxiety measuring factors to assess whether anxiety was situationally determined or relatively independent of the immediate situation (scoring NR) | NR | 🗶 | **1 study** (18) |
| **SAS** | A 20-item tool to assess anxiety scored on a four-point scale (score: 1-4) | 20-80 | 🗶 | **1 study** (19) |
| **Depression PROMs** | | | | |
| **HAM-D** | A 17-item tool for evaluating depression scored on varying scales by item (score: NA) | 0-53 | 🗶 | **4 studies** (6, 7, 21, 23) |
| **BDI** | A 21-item tool which evaluates the severity of depressive symptoms scored on a four-point scale (score: 0-3) | 0-63 | 🗶 | **3 studies** (21, 24, 25) |
| **HADS-D** | A seven-item tool developed to assess psychological distress in non-psychiatric patients scored on a four-point scale (score: 0-3) | 0-21 | 🗶 | **3 studies** (4, 17, 22) |
| **CES-D** | A 20-item tool to assess mood, somatic complaints, and motor inhibition during the last week scored on a four-point scale (score: 0-3) | 0-60^†^ | 🗶 | **1 study** (15, 16) |
| **MDI** | A 10-item tool used as a diagnostic tool for depression scored on a six-point scale (score: 0-5) | 0-50 | 🗶 | **1 study** (10) |
| **PHQ-9** | A 9-item tool for depression in the primary care setting scored on a four-point scale (score: 0-3) | 0-27 | 🗶 | **1 study** (26) |
| **SDS** | A 20-item tool to assess depression scored on a four-point scale (score: 1-4) | 20-80 | 🗶 | **1 study** (19) |
| **Fatigue PROMs** | | | | |
| **CFQ** | An 11-item tool to measure the severity of physical and mental fatigue scored on a four-point scale (score: 0-3) | 0-33 | 🗶 | **2 studies** (17, 22) |
| **FSS** | A 9-item tool estimating the fatigue severity in different situations during the past week scored on a seven-point scale (score: 1-7) | 1-7 (mean of the nine items) | 🗶 | **2 studies** (23, 27) |
| **CIS-f** | An eight-item tool to measure experienced fatigue during the past 2 weeks scored on a seven-point scale (score: 1-7) | 8-56 | 🗶 | **1 study** (28) |
| **FAS** | A 10-item tool to measure physical and mental fatigue scored on a five-point scale (score: 1-5) | 10-50 | 🗶 | **1 study** (24) |
| **FIS** | A 40-item tool to evaluate the impact of fatigue on cognitive, physical, and psychosocial aspects over the last month (scoring NR) | 0-40 (physical and cognitive FIS); 0-80 (psychosocial FIS) | 🗶 | **1 study** (24) |
| **FSMC** | A 20-item tool for assessment of cognitive and physical fatigue scored on a five-point scale (score: 1-5) | 20-100 | 🗶 | **1 study** (15, 16) |
| **MFI-20** | A 20-item tool that measures fatigue severity and categorises fatigue into five domains (general fatigue, physical fatigue, reduced activity, reduced motivation, and mental fatigue) scored on a five-point scale (score: 1-5) | 20-100 | 🗶 | **1 study** (10) |
| **Sleep PROMs** | | | | |
| **ESS** | An eight-item tool to assess subjective daytime sleepiness scored on a four-point scale (score: 0-3) | 0-24 | 🗶 | **4 studies** (19, 23, 24, 29) |
| **PSQI** | A seven-item tool to evaluate sleep quality during the previous month scored on a four-point scale (score: 0-3) | 0-21 | 🗶 | **4 studies** (15, 16, 19, 23, 29) |
| **ISI** | A seven-item tool assessing the subjective symptoms and consequences of insomnia scored on a five-point scale (score: 0-4) | 0-28 | 🗶 | **1 study** (22) |
| **SSA** | A tool consisting of four parts to assess sleep and awakening quality; sleep quality, awakening quality, somatic complaints, and subjective estimates of total sleep time, sleep latency, sleep efficiency and number of nocturnal awakenings (scoring NR) | NR | 🗶 | **1 study** (19) |
| **Disease-specific PROMs** | | | | |
| **MG QoL-15** | A 15-item patient-reported disease-specific QoL questionnaire, consisting of a five-point scale indicating the patient’s agreement with a given statement about MG involvement scored on a five-point scale (score: 0-4) | 0-60 | 🗶 | **10 studies** (7, 10, 12, 15-17, 23, 29-32) |
| **MG-ADL** | An eight-item, clinician-directed but patient-reported questionnaire that assesses common MG symptoms and dysfunctions scored on a four-point scale (score: 0-3) | 0-24 | 🗶 | **9 studies** (10, 15-17, 30, 31, 33-36). |
| **MGFS** | A 26-item tool based on three subscales of fatigue; perception, task avoidance behaviour, and physical exhaustion (scoring NR) | 25-130 | 🗶 | **1 study** (15, 16) |
| **Subjective patient evaluation** | The first item of the IMGQ: the patient had to answer excellent, fine, or poor to the question: ‘‘In general, you would grade your health as:’’. | NA | NA | **1 study** (37) |

Abbreviations: AIS, Acceptance of Illness Scale; BAI, Beck anxiety inventory; BDI, Beck depression inventory; CES-D, Center for Epidemiologic Studies Depression Scale; CFQ, Chalder Fatigue Scale; CIS-f, Checklist Individual Strength fatigue; EORTC QLQ, European Organization for Research and Treatment of Cancer quality of life questionnaire; EQ-5D, EuroQol five-dimensions questionnaire; ESS, Epworth Sleepiness Scale; ESSI-D, ENRICHED Social Support Inventory; FAS, fatigue assessment scale; FIS, fatigue impact scale; FSMC, Fatigue Scale for Motor and Cognitive Functions; FSS, fatigue severity scale; gMG, generalised myasthenia gravis; HADS-A, Hospital Anxiety and Depression Scale Anxiety; HADS-D, Hospital Anxiety and Depression Scale Depression; HAM-A, Hamilton anxiety rating scale; HAM-D, Hamilton depression rating scale; IPAT, Institute for Personality and Ability Testing; ISI, Insomnia Severity Index; MDI, major depression inventory; MFI-20, multidimensional fatigue inventory; MSPSS, Multidimensional Scale of Perceived Social Support; NA, not applicable; NR, not reported; PAIS-SR, Psychosocial Adjustment to Illness Scale; PASS, Patient Acceptable Symptom State; PHQ-9, Patient Health Questionnaire-9; POMS, Profile of Moods Score; PROM, patient reported outcome measure; PSQI, Pittsburgh Sleep Quality Index; PTGI, Post-Traumatic Growth Inventory & Scale; QLI, Quality of Life Index; QoL, quality of life; SAS, Self-Rating Anxiety Scale; SDS, Self-Rating Depression Scale; SF-36, 36-Item Short Form Health Survey; SSA, self-rating questionnaire for sleep and awakening quality; VAS, visual analogue scale.

† Reported as 0-45 in (15, 16).

Supplementary Table 4: Summary of PROM data from the studies included in the systematic review

| Study,  country | Study design (time period) | Total study population | gMG, n (%) | Outcomes | Author conclusions |
| --- | --- | --- | --- | --- | --- |
| **Akkan Suzan 2022** (24), Turkey | Case-control (December 2018 – June 2019)  Outpatient clinic of Neuromuscular Diseases | Consecutive patients aged 18-70 years with confirmed diagnosis of MG (n=53)  Age-matched healthy controls (n=53) | 30 (56.6%)^†^  Criteria: MGFA IIA | FAS, mean (SD): 23.73 (6.77)  FIS total, mean (SD): 32.10 (26.60)  BDI, mean (SD): 10.86 (10.32)  ESS, mean (SD): 4.66 (3.46) | When patient groups and control groups were compared in terms of fatigue, depression and sleepiness, the patient group had higher scores in all scales. In the patient group, the mean values of FAS, FIS physical and BDI were significantly higher than the control group (p=0.003, p=0.001, and p=0.003, respectively). FIS total, FIS cognitive, FIS social, and ESS average scores indicated no significant differences between the two groups.  The mean values of FIS total and FIS cognitive were found to be significantly higher in gMG compared to oMG (p=0.033 and p=0.045), and no significant difference was found between the two groups in terms of FIS physical, FIS social, FAS and depression and sleepiness scales. |
| **Alanazy 2019** (26), Saudi Arabia | Cross-sectional (March 2018 – March 2019)  Two tertiary hospitals (King Saud University Medical City and Security Forces Hospital) in Riyadh, Saudi Arabia | Adult patents with MG (n=104) | 82 (78.8)^†^  Criteria: author reported | PHQ-9, n/N:   - Score <10 (none & mild depression), 59/82 - Score ≥10 (moderate & severe depression, 23/82 | No significant difference was reported in PHQ-9 scores between patients with gMG and oMG (p=0.35). |
| **Ambrogi 2012** (1), Italy | Retrospective cohort (1980-2005)  Tor Vergata University, Rome | Patients with class III AChR+ nonthymomatous MG who underwent transsternal extended thymectomy (n=59) | 59 (100%)^†^  Criteria: MGFA III | SF-36^¶^, PCS ≥50, %:   - Baseline, 22 - 1 year, 25 - 2 years, 29 - 3 years, 34 - 5 years, 43 - 10 years, 61   SF-36^¶^, MCS ≥50, %:   - Baseline, 28 - 1 year, 30 - 2 years, 35 - 3 years, 39 - 5 years, 50 - 10 years, 61 | All domains except body pain significantly improved with time, up to a maximum of 10 years.  At baseline, PCS values were more compromised in the presence of sole skeletal muscle involvement whereas MCS was significantly decreased in patients with oropharyngeal symptoms.  SF-36 PCS and MCS stably achieved the endpoint value in approximately one quarter of patients, and this improved with time. |
| **Andersen 2021** (10), Denmark | Cross-sectional (October 2019 – June 2020)  Copenhagen Neuromuscular Center | Patients aged ≥18 years with a verified diagnosis of gMG, and active medical treatment for MG (n=100) | 100 (100%)^†^  Criteria: author reported | MFI-20, median (IQR):  General fatigue,   - All, 12 (9-16) - PASS-, 17 (12-18) - PASS+, 10 (7-14)   Physical fatigue,   - All, 13 (10-16) - PASS-, 17 (14-18) - PASS+, 11 (9-14)   Reduced activity,   - All, 11 (6-14) - PASS-, 13 (12-16) - PASS+, 9 (6-12)   Reduced motivation,   - All, 8 (6-11) - PASS-, 9 (7-13) - PASS+, 7 (5-10)   Mental fatigue,   - All, 8 (5-12) - PASS-, 11 (7-13) - PASS+, 8 (5-11)   MDI, median (IQR):   - All, 10 (5-18) - PASS-, 17 (12-25) - PASS+, 8 (3-14)   EQ-5D-3L, mean (SD):   - All, 0.8 (0.2) - PASS-, 0.71 (0.66-0.78) - PASS+, 0.83 (0.78-1.00)   EQ-5D-VAS, median (IQR):   - All, 75 (65-80) - PASS-, 60 (50-75) - PASS+, 80 (70-85)   PASS-, %: 33  PASS+, %: 67  MG-ADL, median (IQR):   - All, 3 (0-5) - PASS-, 5 (4-7) - PASS+, 1 (0-3)   MG-QoL 15, median (IQR):   - All, 8 (3-21) - PASS-, 25 (12-32) - PASS+, 6 (1-12) | PASS- patients had significantly higher scores on all outcome measures (p<0.05). |
| **Aysal 2013** (21), Turkey | Cross-sectional (NR)  Istanbul Bakirkoy Neuropsychiatry Teaching and Research Hospital Neuromuscular Diseases Outpatient Clinic | Consecutive patients aged ≥18 years with MG (n=42) | 36 (86)^†^  Criteria: Osserman IIA-IIB | BDI, mean (SD):   - Stage IIA, 14.9 (10.6) - Stage IIB, 18.5 (13.8)   BAI, mean (SD):   - Stage IIA, 7.8 (4.9) - Stage IIB, 13.1 (6.0)   HAM-D, mean (SD):   - Stage IIA, 14.2 (6.9) - Stage IIB, 21.4 (10.1)   HAM-A^¶^, mean (SD):   - Stage IIA, 17.4 (7.9) - Stage IIB, 26.3 (10.1) | Patients with stage IIB MG had significantly higher scores on the BAI, HAM-D, HAM-A total and somatic anxiety than those with stage I and IIA MG (p<0.05).  *This study also provided treatment-comparison which has not been extracted here.* |
| **Bachman 2008** (13), Germany | Prospective cohort (1980 to 2005)  Department for Surgery at the University Medical Center Hamburg-Eppendorf, Germany | Patients with gMG who underwent surgery (n=106) | 106 (100%)^†^  Criteria: Osserman II-IV | EORTC QLQ, median (range):  Physical score,   - Open surgery, 80 (33-100) - Minimally invasive surgery, 80 (0-100) - <45 years, 87 (33-100) - >45 years, 73 (0-100)   Working ability,   - Open surgery, 67 (17-100) - Minimally invasive surgery, 67 (0-100)   Cognitive score,   - Open surgery, 83 (0-100) - Minimally invasive surgery, 67 (0-100) - Stage II, 100 (50–100) - Stage III-IV, 83 (0–100)   Emotional score,   - Open surgery, 83 (25–100) - Minimally invasive surgery, 83 (42-100) - Stage II, 92 (33–100) - Stage III-IV, 75 (25–100)   Social ability,   - Open surgery, 83 (0–100) - Minimally invasive surgery, 83 (0-100) - Stage II, 100 (17–100) - Stage III-IV, 75 (0–100)   Global ability,   - Open surgery, 75 (0-100) - Minimally invasive surgery, 75 (0-100) - <45 years, 83 (33-100) - >45 years, 67 (0-100)   Vegetative score,   - Open surgery, 78 (0-100) - Minimally invasive surgery, 83 (22-100) - <45 years, 83 (11-100) - >45 years, 69 (0-100) - Stage II, 86 (44–100) - Stage III-IV, 72 (0–100) | As expected, QoL was better for younger patients with MG and those with lower Osserman score. |
| **Baram 2021** (33), Iraq | Retrospective cohort (January 2008 – January 2018)  NR | Consecutive patients with non-thymomatous MG who underwent surgery (n=48) | 44 (91.7%)^‡^  Criteria: MGFA IIa-IVb | MG-ADL, mean (SD):  Pre-operative MG-ADL <15, 8.3%  Pre-operative MG-ADL, 15-20, 47.9%  Pre-operative MG-ADL, >20, 43.7%  Pre-operative MG-ADL, 19.92 (4.26)  Post-operative MG-ADL,   - 1 month, 19.66 (2.89) - 3 months, 18.75 (3.98) - 6 months, 15.54 (3.98) - 9 months, 14.07 (3.29) - 12 months, 12.04 (3.55) - 24 months, 9.33 (4.27) - 36 months, 6.67 (4.31) - 48 months, 4.46 (3.71) - 60 months, 2.15 (3.00) | Radical trans-sternal thymectomy for non-thymomatous MG is an effective and well-tolerated procedure associated with improvement in QoL for the majority of patients. |
| **Bartel 1995** (18), South Africa | Case-control (NR)  NR | Patients with MG (n=16)  Matched-control group (n=16) | 15 (93.8%)^‡^  Criteria: author-reported | IPAT anxiety scale, mean (SD):   - A-score, 16.8 (5.9) - B-score, 19.1 (4.3)   POMS, mean (SD):   - Tension, 2.6 (1.0) - Depression, 2.2 (1.0) - Anger, 1.9 90.6) - Fatigue, 3.3 (0.9) - Confusion, 2.4 (0.7) | The MG group revealed significantly higher levels of anxiety, tension, anger, fatigue, and confusion.  Factors such as age, time of onset, severity, duration of MG, sub-type and medication may be relevant to assessment of cognitive and central nervous system function in MG. |
| **Basta 2012** (2), Serbia | Cross-sectional (January 2008 − December 2008)  Outpatient and inpatient settings of the Neurology Clinic, Clinical Centre of Serbia in Belgrade | Consecutive MG patients (n=230) | 120 (52.2%)^†^  Criteria: MGFA IIA-IIIB | *Total SF-36 score is presented by MGFA stage in the publication in Figure 2* | HRQoL in patients with MG is similarly reduced in both psychological and physical aspects.  SF-36 score was shown to decrease with increasing severity of disease. |
| **Birnbaum 2021** (30), France | Case-control (NR)  Data are from a prospective clinical trial (MGEX), prior to exercise intervention | Females with confirmed diagnosis of stable, mild-moderately severe autoimmune gMG without contra-indication to exercise (n=33)  Control subjects (n=66) | 33 (100%)^†^  Criteria: MGFA II-III | MG-ADL total score, median (IQR): 3 (1−4)  MG-QoL 15-F total score, median (IQR): 23 (17−29) | Individuals with stable MG perform less physical activity, at lower intensities, and are more inactive than controls individuals.  Further research is warranted to understand factors influencing physical activity patterns in MG and whether interventions could be successful in increasing physical activity quantity and intensity in individuals with MG. |
| **Busch 1996** (14), Germany | Retrospective cohort (June 1976 – December 1993)  NR | Patients who had undergone transsternal thymectomy for MG (n=65) | NR (94%)^‡^  Criteria: modified Osserman II-IV | Post-operative EORTC, median (10-90% quantile):   - Physical status, 100 (60-100) - Working ability, 100 (50-100) - Cognitive, 100 (33-100) - Emotional, 84 (42-100) - Social, 100 (50-100) - Global QoL, 75 (50-100) - Vegetative, 84 (50-100) | Restoration of QoL to normal was complete at most recent follow-up with regard to physical status, working ability, and cognitive and social functions.  Some deficits persisted in the emotional and vegetative fields. Patient judgement of global QoL and emotional functioning attracted the lowest post-operative rating.  The physical (-0.57), social (-0.59), and vegetative (-0.55) status closely corresponded to the respective Osserman score. |
| **Cioncoloni 2016** (37), Italy | Cross-sectional (NR)  Clinical Neurology Unit, University Hospital of Sien | Patients aged ≥18 years with MG (n=41) | 29 (70.7%)^†^  Criteria: MGFA IIA-IVB | IMGQ questionnaire, n/N:  Excellent-good HRQoL,   - Stage IIA, 4/7 - Stage IIB, 5/6 - Stage IIIA, 2/4 - Stage IIIB, 3/4 - Stage IVA, 3/7 - Stage IVB, 1/1   Poor HRQoL,   - Stage IIA, 3/7 - Stage IIB, 1/6 - Stage IIIA, 2/4 - Stage IIIB, 1/4 - Stage IVA, 4/7 - Stage IVB, 0/1 | Patients with poor HRQoL showed a significantly lower total IMGQ score, as well as a significantly higher total MG-ADL score.  Proportion of patients with excellent or poor HRQoL was similar regardless of MGFA stage. |
| **De Freitas** **Fregonezi 2006** (3), Spain | Prospective cohort (NR)  Specialised MG clinic attached to hospital’s neurology department | Patients with gMG (n=20) | 20 (100%)^†^  Criteria: Osserman IIA-IIB | SF-36^¶^, mean (SD):   - All, 72 (16) - Male, 64 (3) - Female, 64 (11) - Stage IIA, 70 (13) - Stage IIB, 67 (16) | The results for the following domains were lower than the reference values for all groups: physical functioning, role limitation due to physical problems, and general health perceptions.  Significant differences were found between the results for the whole study group and the reference values in the following domains: physical functioning, role limitations due to physical problems, and general health perceptions.  Scores on the scale measuring vitality tended to be below the reference values for the women and for the IIb subgroup.  Scores for general health perceptions were lower than the reference values in all the groups.  Results for all the remaining groups and domains were higher than or only slightly below the reference values for a healthy population. |
| **De Lapiscina 2012** (29), Spain | Cross-sectional (January 2010 – May 2010)  Outpatient neurology clinic | Patients aged ≥18 years with MG (n=54) | 23 (42.6%)^†^  Criteria: author reported | ESS, mean (SD):   - ESS >10, 30.4% - ESS, 7.2 (4.2)   PSQI, mean (SD):   - PSQI >5, 82.6% - PSQI^¶^, 9.3 (4.5)   MG-QoL 15^§^, mean (SD):   - 19.3 (10.5) | The prevalence of a pathological ESS score was higher among patients with gMG than it was in patients in clinical remission (p=0.02).  No significant differences in the quantitative ESS scores were reported between the generalised, ocular, and remission categories.  A statistically significant difference in pathological PSQI was reported between gMG and remission category (p<0.003).  Patients included in the generalised category had the worst results in the PSQI global score and in the scores of almost all PSQI components. |
| **Dewilde 2022** (11), Belgium, Canada, Germany, Italy, Japan, Spain, UK, and USA | Prospective cohort (NR)  Data using a smartphone/tablet application | Patients with MG (n=617) | NR (NR)^†^  Criteria: MGFA II-V | EQ-5D-5L, mean (IQR):   - Stage II, 0.766 - Stage III, 0.648 - Stage IV, 0.527 - Stage V, 0.360 | MG patients have impaired HRQoL. The severity of the disease, the activities of daily living, their physical and mental health, and the necessity for a caregiver all significantly affect patients' HRQoL. |
| **Happe 2004** (19), Austria | Case-control (NR)  Specialised outpatient clinic, Department of Neurology, University of Vienna | Consecutive patients with MG (n=17)  Healthy controls (n=14) | 16 (94.1%)^‡^ Criteria: Osserman II | ESS, mean (SD) (range): 7.4 (4.3) (2−17)  SDS, mean (SD) (range): 33.8 (7.2) (22−46)  SAS, mean (SD) (range): 33.8 (8.0) (24−52)  QLI, mean (SD) (range): 7.5 (1.2) (5.0−9.2)  PSQI, mean (SD) (range): 6.4 (3.8) (2−15)  SSA^¶^, mean (SD) (range): 31.6 (8.4) (20-62) | Subjective sleep and awakening quality and sleep efficiency were reduced (p<0.05), and the number of nocturnal awakenings (p=0.02) as well as dream recall frequency (p=0.02) were increased in patients with MG compared with controls. |
| **Hoffman 2016** (22), Germany | Cross-sectional (December 2012 – December 2013)  Certified Integrated Center for Myasthenia gravis of the Charité – Universitätsmedizin Berlin | Consecutive patients aged ≥18 years with MG (n=200) | 116 (59.5%)^†^  Criteria: MGFA II-IV | CFQ ≥4, %: 71.6  CFQ-T, mean (SD): 17.5 (6.0)  CFQ-P, mean (SD): 12.1 (4.3)  CFQ-M, mean (SD): 5.4 (2.4)  HADS-D ≥8, %: 24.6  HADS-A ≥8 %: 36.0  ISI ≥10, %: 51.3 | Rates of relevant fatigue were significantly higher in patients with gMG compared with those in pharmacological remission (p<0.001).  gMG patients had significantly higher CFQ scores (Likert scale) in the physical as well as in the mental domain compared to remitted MG as well as oMG patients.  Both, mood and sleeping disorders were more frequent in patients with gMG compared to those with oMG or in pharmacological remission. |
| **Jastrezebska 2019** (34), Poland | Cross-sectional (NR)  Neuromuscular center, the Medical University of Warsaw | Patients with JMG (n=101) | 87 (86.1%)^‡^  Criteria: MGFA | MG-ADL, mean (SD): 2.5 (3.1)  MG-ADL, median, (IQR) (range): 1, (4) (0−15) | The treatment outcome for JMG is favourable, with a marked reduction of symptoms and good day-to-day activity achieved for most patients. |
| **Jordan 2017** (15, 16), Germany | Case-control (NR)  Department of Neurology, Martin Luther University Halle | Patients with gMG (n=33)  Healthy controls (n=17) | 33 (100%)^†^  Criteria: MGFA | CES-D, mean (SD, SE): 12.5 (9.6, 1.7)  PSQI, mean (SD, SE): 7.9 (4.9, 0.9)  FSMC, mean (SD, SE): 54.1 (22.7, 3.9)  VAS, mean (SD, SE): 7.1 (2.1, 0.4)  MGFS, mean (SD, SE): 58.8 (22.8, 4.0)  MG-ADL, mean (SD, SE): 3.3 (3.1, 0.5)  MG-QoL 15, mean (SD, SE): 15.7 (13.8, 2.6) | Fatigue and cognitive scores were significantly higher in MG patients than in control subjects (P < 0.001). |
| **Kotan 2016** (4), Turkey | Cross-sectional (NR)  Psychiatry and Neurology Departments of Uludag University Medical Faculty Hospital | Patients with MG (n=52) | 43 (82.7%)^‡^  Criteria: author reported | HADS-D, mean (SD):   - With psychiatric disorders, 9.9 (4.8) - Without psychiatric disorders, 4.3 (3.2)   HADS-A, mean (SD):   - With psychiatric disorders, 10.6 (4.0) - Without psychiatric disorders, 4.5 (2.3)   PAIS-SR, mean (SD):   - With psychiatric disorders, 48.7 (18.9) - Without psychiatric disorders, 36.3 (13.1)   MSPSS^¶^, mean (SD):   - With psychiatric disorders, 59.3 (11.9) - Without psychiatric disorders, 67.7 (10.9)   PTGI, mean (SD):   - With psychiatric disorders, 50.6 (15.9) - Without psychiatric disorders, 53.3 (14)   SF-36^¶^, mean (SD):  Physical functioning   - With psychiatric disorders, 62.1 (16.7) - Without psychiatric disorders, 65.6 (17.2)   Physical role   - With psychiatric disorders, 53.6 (13.6) - Without psychiatric disorders, 57.1 (13.6)   Bodily pain   - With psychiatric disorders, 52.7 (17.9) - Without psychiatric disorders, 55.8 (15.4)   General health   - With psychiatric disorders, 44.1 (11.5) - Without psychiatric disorders, 55 (11.2)   Vitality   - With psychiatric disorders, 39.7 (12.5) - Without psychiatric disorders, 47.4 (12.2)   Social functioning   - With psychiatric disorders, 57.9 (16.9) - Without psychiatric disorders, 68.7 (13.9   Emotional role   - With psychiatric disorders, 61.3 (15.9) - Without psychiatric disorders, 69.2 (13.8)   Mental health   - With psychiatric disorders, 56.9 (12.6) - Without psychiatric disorders, 66.4 (12.2) | Patients with a psychiatric diagnosis had lower scores in general health, vitality, social functioning, emotional role, and mental health domains of SF-36.  PAIS-SR scores, total MSPSS and friends scores were significantly higher in patients without a psychiatric diagnosis.  Significant correlations of psychosocial factors with QoL were determined. |
| **Lehnerer 2021** (17), Germany | Cross-sectional (May 2019 – July 2019)  Members of the German Myasthenia Association | Patients with MG (n=1660) | 1127 (69.6%)^†^  Criteria: author reported | HADS, median (IQR): 11 (6-18)  HADS-A ≥8, %: 36.1  HADS-D ≥8, %: 31.9  CFQ, median (IQR): 18 (13-22)  CFQ ≥4, %: 72.5  ESSI-D ≤18, %: 24.4  MG-ADL, median (IQR): 4 (2-7)  MG-QoL 15, median (IQR): 16 (5-28) | PROMs were worse in patients with gMG compared with oMG patients. |
| **Padua 2001** (5), Italy | Prospective cohort (October 1999 – April 2000)  NR | Patients with MG (n=46) | 44 (95.7%)^‡^  Criteria: Osserman II-IV | SF-36^¶^, mean (SD):   - PCS, 36.0 (10.1) - MCS, 33.2 (9.3) | The Osserman scale and clinical examination findings were significantly related to the physical aspects of HRQoL.  Mental aspects of the QoL were not progressively involved as muscle deficit progressed, but even in a mild clinical picture, the mental aspects were deteriorated.  Patient-oriented measures proved that the patient’s QoL was impaired especially with regard to physical aspects.  *This study also provided treatment-comparison which has not been extracted here.* |
| **Peres 2017** (12), Portugal | Retrospective cohort (February 2010-September 2015)  Neurology clinic and Autoimmune Diseases Unit, Fernando Fonseca Hospital | Patients with gMG treated with rituximab (n=6) | 6 (100%)^†^  Criteria: author reported | Mean difference between pre-treatment (recorded retrospectively) and post-treatment:   - EQ-5D: +0.492 - EQ-5D VAS: +48.3 - MG-QoL 15: -20 | There was a positive tendency towards an improvement in QoL after rituximab treatment. |
| **Raggi 2010** (9), Italy | Prospective cohort (NR)  Besta Institute’s Department of Neuroimmunology and Neuromuscular Diseases | Consecutive adult patients with MG (n=102) | 48 (47.1%)^†^  Criteria: MGFA II-IV | Proportion of patients in cluster groups, n/N:  Cluster A (patients with lower levels of disability, as assessed by WHO-DAS II, and lower QoL decrement, as assessed by SF-36),   - Stage II, 6/51 - Stage III, 6/51 - Stage IV, 5/51   Cluster B (patients with intermediate levels of disability and QoL decrement),   - Stage II, 5/28 - Stage III, 2/28 - Stage IV, 3/28   Cluster C (patients with higher levels of disability and QoL decrement),   - Stage II, 2/23 - Stage III, 3/23 - Stage IV, 16/23 | 66.7% of patients in cluster A were in pharmacological remission or MGFA stage I (oMG). These patients’ HRQoL levels were comparable to those of the general healthy population.  Cluster B mainly consisted of patients belonging to MGFA stage I (oMG).  Cluster C was mainly composed of patients in stage IV (69.6%). |
| **Rodolico 2021** (35), Italy | Retrospective cohort (NR)  University of Messina, Italy | Patients with gMG treated with methotrexate (previously treated with azathioprine for at least 12 months) (n=15) | 15 (100%)^†^  Criteria: MGFA II-III | *MG-ADL score decrease (improvement) over time is presented in the publication in Figure 2* | MG-ADL score decreased significantly on each follow-up visit. |
| **Roth 2002** (20), Switzerland | Retrospective cohort (1986-1989)  NR | Consecutive patients that underwent transsternal radical thymectomy for gMG (n=23) | 23 (100%)^†^  Criteria: Osserman II-IV, Oosterhuis classification | Subjective patient evaluation of QoL, n:   - Very good condition, 6 - Good condition, 4 - Better than before surgery, 9 - No change, 3 - Worse than before surgery, 1   Able to perform sports regularly, n: 14 | Transsternal thymectomy was associated with an improvement to patient QoL. |
| **Ruckert 2003** (36), Germany | Retrospective cohort (NR)  Humboldt University Medical School | Patients who underwent thymectomy for gMG (n=182) | 182 (100%)^†^  Criteria: Osserman II-III | Cumulative MG-ADL improvement from before thymectomy to last follow-up, median (range):   - tThx, 6.0 (1-19) - sThx, 5.5 (2-14) - aThx, 7.5 (0-12 | There was no significant difference for ADL measurements between the three approaches for thymectomy. |
| **Ruiter 2021** (28), Belgium | Cross-sectional (NR)  Dutch-Belgian Myasthenia Patient Registry | All Dutch patients with MG from the Dutch-Belgian Myasthenia Patient Registry (n=420) | NR (NR)^†^  Criteria: positive scores on MG-ADL item(s) 5 and/or 6 | CIS-f, mean (SD):   - Overall, 41.7 (10.3) - Female, 43.7 (9.3) - Male, 38.7 (11.0) | The mean fatigue score was significantly higher in patients with generalised symptoms compared to those without clinical symptoms (p<0.001).  Women had significantly higher fatigues cores compared to men within all MG-ADL subgroups except oculobulbar symptoms. |
| **Sabre 2017** (27), Estonia | Cross-sectional (NR)  Tartu University Hospital | Patients with confirmed MG diagnosis in Estonian cohort (n=36) | 29 (80.6%)^†^  Criteria: author reported | FSS, mean (SD):   - Estonia, 5.0 (1.7) - Sweden, 3.4 (2.3) | Mean FSS was considerably higher in Estonia compared to Sweden (p=0.001). |
| **Sitek 2000** (25), Poland | Case-control (NR)  Myasthenia Gravis Outpatient Clinic | Patients with MG (n=33)  Control group (n=30) | 29 (87.9%)^‡^  Criteria: MGFA II-IV | BDI, mean (SD): 11.10 (6.29) | MG patients exhibited significantly higher BDI scores than control patients (p=0.003). |
| **Stankovic 2018** (6), Serbia | Prospective cohort (June 2012 - February 2013)  Outpatient Unit of the Neurology Clinic, Clinical Center of Serbia | Patients with MuSK MG (n=35)  Patients with AChR MG (n=38) | MuSK: 35 (100%)^†^  AChR: 35 (92.9%)^‡^  Criteria: MGFA IIA-V | HAM-A, mean (SD):   - MuSK, 12.4 (11.2) - AChR, 9.7 (8.1)   HAM-D, mean (SD):   - MuSK, 12.3 (12.1) - AChR, 8.5 (9.4)   MSPSS, mean (SD):   - MuSK, 77.3 (9.3) - AChR, 70.6 (14.1)   AIS, mean (SD):   - MuSK, 19.6 (9.5) - AChR, 20.3 (7.3)   SF-36^¶^, mean (SD):   - MuSK, 64.7 (25.3) - AChR, 52.1 (22.2) | Overall the results showed decreased QoL in patients with MuSK MG.  Physical domain scores of QoL were similarly affected in both MuSK and AChR groups, while mental domain and total SF-36 scores were even better in MuSK MG patients.  Social support was better in the MuSK group (p<0.05). |
| **Stojanov 2019** (7), Serbia | Prospective cohort (January 2017 – 2018)  Clinic of Neurology, Nis | Patients suffering with MG (n=70) | NR (78.6%)^†^  Criteria: MGFA II-IV | *Mean HAM-D, HAM-A, SF-36, and MG-QoL scores are presented in the publication by MGFA stage in Figure 2 and by MG subtype (gMG, oMG or bMG) in Figure 3.* | Patients with severe clinical manifestations and longer duration of the disease, as well as those with anxiety and depression, have poorer QoL.  Patients with the bulbar manifestation of the disease showed lower scores on the QoL questionnaire than patients with gMG or oMG. |
| **Szczudlik 2020** (8), Poland | Cross-sectional (2010-2015)  Department of Neurology in Warsaw Medical University | Adult patents with MG (n=339) | 228 (67.3%)^†^  Criteria: MGFA IIA-IVB | *Mean SF-36 scores are presented by MGFA stage in the publication in Figure 1.* | Higher MGFA score was related to worse QoL in general health (p<0.001), PCS (p<0.001), and MCS (p<0.001) domains. |
| **Tascilar 2018** (23), Turkey | Case-control (NR)  Outpatient clinic of the Neurology Department of Bulent Ecevit University Medical Faculty | Clinically stable patients aged ≥18 years with gMG (n=19)  Healthy controls (n=26) | 19 (100%)^†^  Criteria: MGFA II-III | ESS, mean (SD): 5.1 (5.2)  PSQI, ^¶^, mean (SD): 7.8 (4.5)  FSS, mean (SD): 4.2 (1.9)  HAM-A, mean (SD): 14.5 (8.7)  HAM-D, mean (SD): 15.4 (9.9) MG-QoL 15, mean (SD) [range]: 24.4 (15.4) [0-52] | The scores of HAM-D, HAM-A, FSS and PSQI total scores were significantly higher in patients than controls.  After adjusting for HAM-D and HAM-A scores, FSS and PSQI total scores were not different between patients and controls. |
| **Thomsen 2021** (31), Denmark | Prospective cohort (NR)  Department of Neurology, Aarhus University Hospital | Patients with MG at baseline (n=107)  Patients with MG at follow-up after receiving SOC (n=70) | Baseline: 95 (88.8%)^‡^  Follow-up: 53 (75.7%)^‡^  Criteria: MGFA II-IV | MG-ADL, mean (95% CI):   - Baseline, 3.5 (2.97-4.04) - Follow-up, 2.3 (1.76-2.74)   MG-QoL 15, mean (95% CI):   - Baseline, 11.5 (9.46-13.56) - Follow-up, 8.5 (6.66-10.32) | Patients improved on all clinical scores: QMG -1.8 (p< .001), MGC-1.5 (p< .001), MG-ADL -1.3 (p< .001), and MG-QoL 15 -3.0 (p< .001).  Females improved less than males on the QMG (p=.01), MGC (p< .001), MG-ADL (p=.006), and MG-QoL 15 (p< .001) independent of potential confounders. |
| **Westerberg 2018** (32), Sweden | Cross-sectional (2010-2014)  Departments of Neurology at hospitals in Jönköping, Eksjö or Värnamo | Adult patients with ICD-10 codes G70.0 or G70.9 (n=40) | 31 (77.5)^†^  Criteria: author reported | MG-QoL 15, median (range): 15.5 (0-42) | Patients with current generalised MG scored higher on MG-QoL 15 than patients without current generalised symptoms (p<0.0001). |

Abbreviations: AChR, acetylcholine receptor; ADL, activities of daily living; AIS, Acceptance of Illness Scale; aThx, thymectomy through anterolateral thoracotomy; BAI, Beck anxiety inventory; BDI, Beck depression inventory; CES-D, Center for Epidemiologic Studies Depression Scale; CFQ, Chalder Fatigue Scale; CFQ-M, Chalder Fatigue Scale Mental; CFQ-P, Chalder Fatigue Scale Physical; CFQ-T, Chalder Fatigue Scale Total; CIS-f, Checklist Individual Strength fatigue; EORTC QLQ, European Organization for Research and Treatment of Cancer quality of life questionnaire; CI, confidence interval; EQ-5D, EuroQol five-dimension scale questionnaire; ESS, Epworth sleepiness scale; ESSI-D ENRICHED Social Support Inventory; FAS, fatigue assessment scale; FIS, fatigue impact scale; FSMC, Fatigue Scale for Motor and Cognitive Functions; FSS, fatigue severity scale; gMG, generalised myasthenia gravis; HADS-A, Hospital Anxiety and Depression Scale Anxiety; HADS-D, Hospital Anxiety and Depression Scale Depression; HAM-A, Hamilton anxiety rating scale; HAM-D, Hamilton depression rating scale; IQR, interquartile range; IMGQ, Italian myasthenia gravis questionnaire; IPAT, Institute for Personality and Ability Testing; ISI, Insomnia Severity Index; IQR, interquartile range; JMG, juvenile-onset myasthenia gravis; MCS, mental component summary; MDI, major depression inventory; MFI-20, multidimensional fatigue inventory; MG, myasthenia gravis; MG-ADL, MG Activities of Daily Living Profile; MGFA, Myasthenia Gravis Foundation of America; MGFS, myasthenia gravis fatigue scale; : MG-QoL 15, MG-specific quality of life instrument; mMSPSS, Multidimensional Scale of Perceived Social Support; MuSK, Muscle-specific tyrosine kinase; NR, not reported; oMG, ocular myasthenia gravis; PAIS-SR, Psychosocial Adjustment to Illness Scale; PASS, Patient Acceptable Symptom State; PCS, physical component summary; PHQ-9, Patient Health Questionnaire-9; POMS, Profile of moods score; PROM, patient reported outcome measure; PSQI, Pittsburgh Sleep Quality Index; PTGI, Post-Traumatic Growth Inventory & Scale; QLI, Quality of Life Index; QMG, Quantitative Myasthenia Gravis Clinical Score; QoL, quality of life; SAS, Self-Rating Anxiety Scale; SD, standard deviation; SDS, Self-Rating Depression Scale; SE, standard error; SF-36, 36-Item Short Form Health Survey; SSA, self-rating questionnaire for sleep and awakening quality; sThx, thymectomy through extended median sternotomy; tThx, thoracoscopic thymectomy; VAS, visual analogue scale.

† All outcomes reported for gMG subgroup/population.

‡ Data for one or more outcomes reported for overall population; % gMG ≥80%. No available subgroup data for gMG population.

¶ Further breakdown of individual components of the PROM reported in publication.

References

1. Ambrogi V, Mineo TC. Active ectopic thymus predicts poor outcome after thymectomy in class III myasthenia gravis. J Thorac Cardiovasc Surg. 2012;143(3):601-6.

2. Basta IZ, Pekmezovic TD, Peric SZ, Kisic-Tepavcevic DB, Rakocevic-Stojanovic VM, Stevic ZD, et al. Assessment of health-related quality of life in patients with myasthenia gravis in Belgrade (Serbia). Neurological Sciences. 2012;33(6):1375-81.

3. De Freitas Fregonezi GA, Regiane-Resqueti V, Pradas J, Vigil L, Casan P. The relationship between lung function and health-related quality of life in patients with generalized myasthenia gravis. [Spanish]. Archivos de Bronconeumologia. 2006;42(5):218-24.

4. Kotan VO, Kotan Z, Aydin B, Taskapilioglu O, Karli HN, Yalvac HD, et al. Psychopathology, psychosocial factors and quality of life in patients with myasthenia gravis. Journal of Neurological Sciences. 2016;33(3):482-93.

5. Padua L, Evoli A, Aprile I, Caliandro P, Mazza S, Padua R, et al. Health-related quality of life in patients with myasthenia gravis and the relationship between patient-oriented assessment and conventional measurements. Neurological Sciences. 2001;22(5):363-9.

6. Stankovic M, Peric S, Stojiljkovic Tamas O, Stankovic T, Nikolic A, Lavrnic D, et al. Quality of life in patients with MuSK positive myasthenia gravis. Acta Neurol Belg. 2018;118(3):423-7.

7. Stojanov A, Milosevic V, Dordevic G, Stojanov J. Quality of Life of Myasthenia Gravis Patients in Regard to Epidemiological and Clinical Characteristics of the Disease. Neurologist. 2019;24(4):115-20.

8. Szczudlik P, Sobieszczuk E, Szyluk B, Lipowska M, Kubiszewska J, Kostera-Pruszczyk A. Determinants of Quality of Life in Myasthenia Gravis Patients. Frontiers in Neurology. 2020;11 (no pagination)(553626).

9. Raggi A, Leonardi M, Antozzi C, Confalonieri P, Maggi L, Cornelio F, et al. Concordance between severity of disease, disability and health-related quality of life in Myasthenia gravis. Neurological Sciences. 2010;31(1):41-5.

10. Andersen LK, Jakobsson AS, Revsbech KL, Vissing J. Causes of symptom dissatisfaction in patients with generalized myasthenia gravis. Journal of Neurology. 2021.

11. Dewilde S, Kousoulakou H, Janssen M, Claeys K, Friconneau M, Jacob S, et al. Digital Data Collection to Measure the Impact of Myasthenia Gravis on Patients' Quality of Life in the Real World: Report at Baseline. Value in Health. 2022;25(1 Supplement):S246.

12. Peres J, Martins R, Alves JD, Valverde A. Rituximab in generalized myasthenia gravis: Clinical, quality of life and cost-utility analysis. Porto Biomedical Journal. 2017;2(3):81-5.

13. Bachmann K, Burkhardt D, Schreiter I, Kaifi J, Busch C, Thayssen G, et al. Long-term outcome and quality of life after open and thoracoscopic thymectomy for myasthenia gravis: Analysis of 131 patients. Surgical Endoscopy and Other Interventional Techniques. 2008;22(11):2470-7.

14. Busch C, Machens A, Pichlmeier U, Emskotter T, Izbicki JR. Long-term outcome and quality of life after thymectomy for myasthenia gravis. Annals of Surgery. 1996;224(2):225-32.

15. Jordan B, Schweden TLK, Mehl T, Menge U, Zierz S. Cognitive fatigue in patients with myasthenia gravis. Muscle & nerve. 2017;56(3):449-57.

16. Jordan B, Mehl T, Schweden TLK, Menge U, Zierz S. Assessment of physical fatigability and fatigue perception in myasthenia gravis. Muscle & nerve. 2017;55(5):657-63.

17. Lehnerer S, Jacobi J, Schilling R, Grittner U, Marbin D, Gerischer L, et al. Burden of disease in myasthenia gravis: taking the patient's perspective. Journal of Neurology. 2021.

18. Bartel PR, Lotz BP. Neuropsychological test performance and affect in myasthenia gravis. Acta Neurologica Scandinavica. 1995;91(4):266-70.

19. Happe S, Klosch G, Zeitlhofer J. Perception of dreams and subjective sleep quality in patients with myasthenia gravis. Neuropsychobiology. 2004;50(1):21-7.

20. Roth T, Ackermann R, Stein R, Inderbitzi R, Rosler K, Schmid RA. Thirteen years follow-up after radical transsternal thymectomy for myasthenia gravis. Do short-term results predict long-term outcome? European Journal of Cardio-thoracic Surgery. 2002;21(4):664-70.

21. Aysal F, Karamustafalioglu O, Ozcelik B, Yilmaz M, Karamustafalioglu N, Yumrukcal H, et al. The relationship of symptoms of anxiety and depression with disease severity and treatment modality in Myasthenia gravis: A cross-sectional study. Noropsikiyatri Arsivi. 2013;50(4):295-300.

22. Hoffmann S, Ramm J, Grittner U, Kohler S, Siedler J, Meisel A. Fatigue in myasthenia gravis: risk factors and impact on quality of life. Brain and Behavior. 2016;6(10) (no pagination)(e00538).

23. Tascilar NF, Saracli O, Kurcer MA, Ankarali H, Emre U. Is there any relationship between quality of life and polysomnographically detected sleep parameters/disorders in stable myasthenia gravis? Acta Neurol Belg. 2018;118(1):29-37.

24. Akkan Suzan A, Kahraman Koytak P, Uluc K, Tanridag T. Physical and mental fatigue in myasthenia gravis and its correlation with other symptoms. Acta Neurol Belg. 2022;25:25.

25. Sitek EJ, Bilińska MM, Wieczorek D, Nyka WM. Neuropsychological assessment in myasthenia gravis. Neurological Sciences. 2009;30(1):9-14.

26. Alanazy MH. Prevalence and associated factors of depressive symptoms in patients with myasthenia gravis: A cross-sectional study of two tertiary hospitals in Riyadh, Saudi Arabia. Behavioural Neurology. 2019;2019 (no pagination)(9367453).

27. Sabre L, Westerberg E, Liik M, Punga AR. Diversity in mental fatigue and social profile of patients with myasthenia gravis in two different Northern European countries. Brain and Behavior. 2017;7(4) (no pagination)(e00653).

28. Ruiter AM, Verschuuren JJGM, Tannemaat MR. Prevalence and associated factors of fatigue in autoimmune myasthenia gravis. Neuromuscular Disorders. 2021;31(7):612-21.

29. De Lapiscina EHM, Aguirre MEE, Blanco TA, Pascual IJ. Myasthenia gravis: Sleep quality, quality of life, and disease severity. Muscle and Nerve. 2012;46(2):174-80.

30. Birnbaum S, Bachasson D, Sharshar T, Porcher R, Hogrel JY, Portero P. Free-Living Physical Activity and Sedentary Behaviour in Autoimmune Myasthenia Gravis: A Cross-Sectional Study. Journal of neuromuscular diseases. 2021;8(4):689-97.

31. Thomsen JLS, Vinge L, Harbo T, Andersen H. Gender differences in clinical outcomes in myasthenia gravis: A prospective cohort study. Muscle and Nerve. 2021;64(5):538-44.

32. Westerberg E, Landtblom AM, Punga AR. Lifestyle factors and disease-specific differences in subgroups of Swedish Myasthenia Gravis. Acta Neurologica Scandinavica. 2018;138(6):557-65.

33. Baram A, Salih KAH, Saqat BH. Thymectomy for non-thymomatous myasthenia gravis: Short and long term outcomes, a single-center 10 years' experience. International Journal of Surgery Open. 2021;35 (no pagination)(100381).

34. Jastrzebska A, Jastrzebski M, Ryniewicz B, Kostera-Pruszczyk A. Treatment outcome in juvenile-onset myasthenia gravis. Muscle and Nerve. 2019;59(5):549-54.

35. Rodolico C, Bonanno C, Brizzi T, Nicocia G, Trimarchi G, Lupica A, et al. Methotrexate as a steroid-sparing agent in myasthenia gravis: A preliminary retrospective study. Journal of Clinical Neuromuscular Disease. 2021;23(2):61-5.

36. Ruckert JC, Sobel HK, Gohring S, Einhaupl KM, Muller JM. Matched-pair comparison of three different approaches for thymectomy in myasthenia gravis. Surg Endosc. 2003;17(5):711-5.

37. Cioncoloni D, Casali S, Ginanneschi F, Carone M, Veronica B, Rossi A, et al. Major motor-functional determinants associated with poor self-reported health-related quality of life in myasthenia gravis patients. Neurological Sciences. 2016;37(5):717-23.
